# How can policy and policymaking foster climate justice? A qualitative systematic review

**DOI:** 10.12688/openreseurope.15719.2

**Published:** 2023-10-10

**Authors:** Paul Cairney, Irina Timonina, Hannes Stephan

**Affiliations:** 1 History, Heritage, and Politics, University of Stirling, Stirling, Stirling, FK94LA, UK

**Keywords:** Policy, Policymaking, Climate change, Climate justice, Equity, Just transition

## Abstract

**Background:**

Climate change research has established general requirements for policy and policymaking: transformational changes in policy and policymaking to foster ‘climate justice’, including a ‘just transition’ or movement towards environmental sustainability with equitable processes and outcomes. However, there is a major gap between these requirements and actual policies and policy processes. We identify how researchers use policy theories to understand this gap.

**Methods:**

We conducted a qualitative systematic review (2022) to identify peer reviewed journal articles on climate change, policy, justice, and equity in three databases (Web of Science, Scopus, Proquest). Each article had to provide a non-trivial reference to policymaking concepts or theories. We used an immersive and inductive approach to identify key themes and show how the use of policy concepts and theories informs climate change research.

**Results:**

A total of 108 texts meet the inclusion criteria (with some bias towards Global North research since all texts are in English). Most provide general definitions of climate justice, require fair outcomes and processes, and list what is required to meet those aims. However, they also identify unjust processes and outcomes in relation to who is recognised, gets to define the problem, and wins or loses from solutions. Researchers contrast their preferred social justice approach (informing ‘civic environmentalism) to a dominant neoliberal approach (corresponding to weak ‘ecological modernization’).

**Conclusions:**

Researchers focus on what they need from policy and policymaking to produce climate justice. Few engage meaningfully with policy theories to describe how policymaking actually works. More engagement would help to set meaningful expectations regarding policy change and avoid a needless tendency to treat policymaking like a ‘black box’.

## Plain language summary

There is a strong and coherent message in environmental research: climate change represents an urgent global crisis. Although it is everyone’s problem, there are major inequalities regarding who causes it and who suffers its impact the most. These problems relate not only to geography but also factors such as income, gender, and race. Governments need to address climate change by transitioning to sustainable energy, transport, food, and other systems. They need to ensure climate justice by fostering inclusive policy processes (who is heard, and who defines the problem?) plus equitable contributions (who pays to solve the problem?) and outcomes (who wins or loses?). They also need to collaborate to create fair policy processes that produce both transformational and equitable policy change.

However, this research struggles to explain the gap between these requirements versus actual policies and policymaking. To some extent, the cause seems obvious: there is too much empty talk by powerful political actors, and too little motivation or political will to do the right thing. Yet, policy theories show that these gaps also have systemic causes that would persist even when addressed by sincere and energetic policymakers.

Therefore, our objective is to identify how environmental researchers engage with policy theories to better understand and address this problem. Generally speaking, studies criticise the dominance of one approach that rejects radical change and puts too much faith in market mechanisms and technological innovation. Few studies use policy theories to identify how policy processes could facilitate a radically new approach. More conceptual engagement would help to set meaningful expectations for policy change and avoid a tendency to treat policymaking like a mysterious ‘black box’ rather than a well-studied process.

## Introduction

We present a qualitative systematic review of climate change policy research, focusing on the study of climate justice or just transitions to sustainability. Our review suggests that climate change research has three general requirements for policy and policymaking:

- First, transformational policy change to mitigate climate change and adapt to its increasingly severe impacts. In other words, to transition from unsustainable to sustainable systems to generate and use energy, transport people, produce food, maintain homes, and consume goods and services.
- Second, since climate change transcends national borders, governments need to collaborate with each other – and many actors inside/outside of government - to produce rapid and radical policy change.
- Third, a ‘just transition’ towards environmental sustainability requires fair policy processes and equitable outcomes (broadly speaking, most accounts use justice or equity interchangeably to describe fairness).

Our review suggests that current research highlights a major gap between these requirements and actual policies and processes. For example, policy responses are not proportionate to the size of the problem: the aim of a just transition is low profile and ignored, or intermittently high profile and contested. Overall, we highlight an increasingly worrying climate crisis with unequal and unfair consequences. For example, we identify a general narrative that some people, organisations, and countries are disproportionately responsible for the actions that exacerbate climate change, while others shoulder an unequal burden. This inequity relates to current spatial injustice and intergenerational injustice, as well as: the unequal recognition of the right to participate in policy processes which marginalizes many relevant voices; unfair policy processes that privilege some actors; and, outcomes that exacerbate marginalization in relation to factors such as poverty, gender, race, and disability. Our review suggests that these injustices are apparent in climate change mitigation (preventing or reducing the impact of climate change) *and* adaptation (adjusting to its effects).

In that context, our objective is to identify how researchers use policy concepts and theories to understand and address this multifaceted problem. Previous review articles have contributed separately to key elements of this task, to identify how climate researchers: conceptualise justice or injustice ( Malloy & Ashcraft, 2020; Pellegrini-Masini *et al*., 2020; Sokołowski & Heffron, 2022), establish feasible pathways to policy change ( Sovacool *et al*., 2020; Werners *et al*., 2021), describe the injustices that can result from climate-related policy changes ( Koch & Verholt, 2020; Sovacool, 2021), identify the policy concepts and theories useful to climate research ( Huitema *et al*., 2016), and use key theories as part of a framework to assess climate governance ( Burch *et al*., 2019). Here, we consolidate and build on such work to provide an overall assessment of the use of policy concepts and theories to inform research on climate justice policy and policymaking.

This objective relates to a wider aim to map the use of policy concepts and theories to understand and address inequity in multiple policy sectors, focusing on intersectoral initiatives (often described as ‘mainstreaming’ or ‘integration’) since problems such as inequalities transcend traditional policy departments. To that end, we designed this study as a partner to three other reviews of research in relation to equity and: public health (Health in All Policies, HiAP) ( Cairney *et al*., 2021), education ( Cairney & Kippin, 2022), and gender mainstreaming policies ( Cairney *et al*., 2022a; the full review is in progress).

We seek to learn from each review rather than replicate the same model uniformly. We are flexible enough to identify key differences in each field, such as regarding terminology, guiding assumptions, reference points, and the country of the researcher or their object of study. An immersive and inductive approach to this data allows us to generate a collection of themes that are, to some extent, particular to each field. In that context, it is striking that very similar themes emerge in climate justice research.

First, climate justice researchers provide a similar narrative on the context for research. They identify the urgency and importance of climate change, and how it relates to widespread and unfair inequalities in the economy and society. These inequalities are manifest in relation to vulnerability to the effects of climate change *and* the attempts by governments to mitigate and adapt to climate change. Further, while most research recognises the role of the IPCC (Intergovernmental Panel on Climate Change) in establishing the evidence for climate change, and of the UNFCCC (United Nations Framework Convention on Climate Change) in coordinating an international response, there is some scepticism about either organisation’s engagement with climate justice as a distinct concern.

Second, there is an intense competition to define the problem and identify solutions. Most studies identify high political contestation to decide what justice, fairness, or equity mean, and how those definitions should relate to policy. Although there is a wide range of context-specific applications, most studies highlight the need to foster three kinds of social justice:

- *Recognitional*, to challenge the privileging of some voices and marginalisation of others.
- *Procedural*, to ensure fair ways to participate, deliberate, inform and make choices.
- *Distributional*, to ensure fair ways to pay for, and minimise inequalities associated with, climate change mitigation and adaptation.

Then, they contrast their preferred social justice approaches with neoliberal approaches, characterised by attempts to (1) prioritise economic growth over other aims such as sustainability and reducing inequalities, and favour market-based interactions over state responsibility and interventionism, often while (2) trying to ‘depoliticise’ climate change and restricting input to scientific or technocratic experts.

Third, they identify the transformational changes to policy and policymaking that are required to foster climate change mitigation and adaptation *and* ensure social justice. This agenda includes the ‘mainstreaming’ of environmentalism across (and outside of) government, the design of effective and equitable policy tools, and a more general challenge to existing approaches that depoliticise the problem and put too much faith in markets and technological innovation.

Fourth, they contrast such requirements with actual policies and processes. Most studies describe the dominance of neoliberal approaches, or reliance on a very weak version of ‘ecological modernization’ favouring modest state intervention, which hinders proportionate policy change in line with social justice. They also describe top-down and exclusionary policy processes that produce unfair results. This problem can include policy changes in principle that are not delivered in practice, such as when high-level climate change commitments meet with business-as-usual policymaking, or when environmental schemes are beset by unequal participation and unfair outcomes.

Finally, they recognize the need to use policymaking research to understand the processes that constrain or facilitate transformational change. Compared to our health review, few use policy theories instrumentally to seek practical lessons, such as how to advocate for policy change or encourage greater cooperation across levels of government and policy sectors. Rather, as with our education review, most try to pinpoint the actors and discourses that are getting in the way of progress.

However, our Discussion section reflects on the generally low engagement with mainstream policy theories in this field of study. We argue that climate justice research is incomplete without a full appreciation of the policy processes that constrain or facilitate policy change. In particular, we describe three core policy theory insights that should guide interdisciplinary research: (1) major policy change is rare and difficult to predict; (2) policymaking is subject to ‘bounded rationality’ which prompts policymakers to ignore most policy problems (and evidence) and reject most ways to interpret them; and, (3) policymakers operate ‘in a complex policymaking environment of which they have limited knowledge and control’ ( Cairney & Kippin, 2022: 3). Without such insights, researchers may treat the climate policy process as a ‘black box’, not fully understand a lack of policy progress, or misdiagnose the problem and address it ineffectively ( Biesbroek *et al*., 2015; Morrison *et al*., 2019).

## Methods

We adapt Kuckertz and Block’s (2021) guidance on describing systematic reviews with the following categories.

*Rationale*. We seek to understand the use of policy concepts and theories in climate justice research. These theories are essential to research that (1) identifies requirements for policy and policymaking change, but (2) is unable to fully appreciate how policy processes work. Our reviews are part of the Horizon 2020 project *Integrative Mechanisms for Addressing Spatial Justice and Territorial Inequalities in Europe* (IMAJINE) (Cairney is Co-Investigator). It aims to identify how policymakers and researchers understand ‘spatial justice’ and seek to reduce ‘territorial inequalities’. Our role is to examine how ‘(a) policy actors compete to define the policy problem of equity or justice in relation to inequalities’, and (b) ‘identify priorities in relation to factors such as geography, gender, class, race, ethnicity, and disability’ ( Cairney & Kippin, 2022: 5).

*Engagement with previous reviews*. This is our third qualitative systematic review submitted to Open Research Europe. The first review on health equity ( Cairney *et al*., 2021) sought lessons from studies of the use of policy theories in other disciplinary or interdisciplinary fields ( Embrett & Randall, 2014; Munro & Cairney, 2020). Such *et al*. (2019) guided our first protocol.

*Research/ guiding questions*. Each review has the same guiding questions, and the following discussion draws heavily on the Methods section of Cairney *et al*. (2021: 4–6) and Cairney and Kippin (2022: 5–8). It begins with our general focus:

- **What is the policy problem?** How do researchers define equity, and identify what constrains or facilitates its progress?
- **How does it relate to policy processes?** Do articles identify a lack of policy progress and how to address it? What policy theories do they use when describing policymaking?

Each review’s overall guiding question is:

- How does equity research use policy theory to understand policymaking?

The guiding question for article inclusion is:

- How many studies provide a non-trivial reference to policymaking concepts or theories?

Additional questions to guide analysis have included, for example, what transferable lessons do these studies provide? However, we have found in previous reviews that most articles do not provide substantive answers to such questions. In that context, Cairney *et al*. (2022b) draw on a wider pool of conceptual and empirical studies to answer those question in relation to territorial politics, inequalities, and health, education, and gender mainstreaming strategies. Here, we focus on making sense of climate justice research and its engagement with policy concepts, based more on immersion in the field than trying to stretch the results to fit our previous categories and questions.

*Databases and initial search terms*. We searched three databases ( Web of Science, Scopus, and ProQuest) using broad search terms (climate change AND policy AND justice OR equity). These terms are broadly equivalent to those in health and education, but in a new context with distinctive reference points (which we describe at the beginning of Results). We sought to understand how climate researchers define terms like ‘justice’ or ‘equity’ rather than to include/ exclude based on a single definition. We reached a saturation point by the third database. We learned from previous reviews that the addition of further specialist databases provided minimal additional returns (health), and provided less value than snowballing from the initial set (education).

*Timeliness*. We ran this search from June-October 2022 (last search date was 20.6.22 for Scopus and Web of Science and 13.10.22 for ProQuest).

*Manual searches and choices regarding initial inclusion*. We used similar criteria for inclusion as the other ORE reviews (including publication in English), but with modest changes to reflect our experience with previous searches. First, we allowed for the inclusion of books and book chapters because our review of education highlighted a relatively high reliance on books compared to health (although it made minimal difference to this review). Second, we considered using a stricter interpretation of ‘non-trivial’ to produce an equally manageable number of included articles from a larger set (over 6000 initial entries, compared to over 4000 in education and 5000 in health), but found that the same approach yielded similar results. Each review sets a lower bar for inclusion than other comparable studies, reflecting our confidence that a ‘wide search parameter and low inclusion bar’ helps ‘to generate a broad narrative of the field, identify a sub-set of the most policy theory-informed articles, and examine how the sub-set enhances that narrative’ ( Cairney & Kippin, 2022: 5; compare with the more restrictive approach to energy systems in Munro & Cairney, 2020).

Third, as with the education review, we included articles that: (1) cite policy theories indirectly via reference to the discipline-specific literature (such as ‘political ecology’ described by scholars including Sovacool, or the ‘Earth System Governance’ network) and/or (2) produced relevant work with reference to critical or interpretive approaches to policy discourse (generally with reference to Hajer or Dryzek) or to approaches that would be relevant to our other reviews (such as feminist research), and/or (3) where an insistence on citing mainstream policy theories from the Global North would exclude useful articles produced in the Global South. In other words, we do not exclude texts engaging with anti-mainstream sources when we seek to understand engagement with mainstream policy theories. These choices helped to secure a substantive coverage of key themes - such as the competition between ‘ecological modernization’ (EM) and ‘civic environmentalism’ (CE) – and highlight (albeit in a small number of cases) the mildly blurry boundary between inclusion and exclusion that should be expected in qualitative research. However, they do not guarantee a comprehensive review of climate justice research. For example, Brandstedt’s (2023) peer review notes that our search rules – requiring engagement with policy concepts - lead to the omission of ‘the field of study known as "climate justice" or "climate ethics" to which normative political theorists and philosophers have contributed since the early 1990s’. Further, we confirm, after reviewing the sample of texts described by Brandstedt (2023), that such research would generally not be included in our review. For example, Caney (2021) does not discuss policymaking directly or refer to any concepts or sources described in our list. We examine this omission as part of the Limitations section. Nor does our wide inclusion solve more fundamental issues regarding how to *synthesise* insights while using texts that reproduce different academic traditions. While Durnova and Weible (2020) provide an optimistic take on reconciling mainstream (often described as ‘positivist’) and interpretive (post- or anti-positivist) approaches to policy research, the authors themselves may not agree (e.g. Hajer, 1995: 68–72).

**Figure 1.**
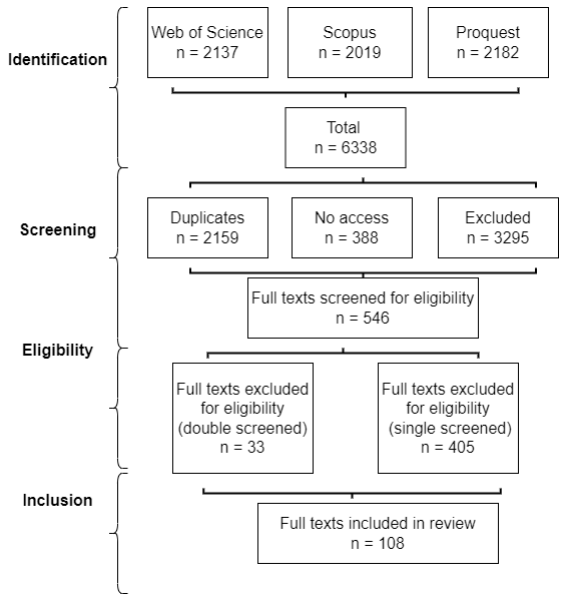
Review process flow chart.

Timonina conducted a manual search of the full text to find articles that made at least one reference to an established policy theory (such as multiple streams or the advocacy coalition framework) or concept (such as new institutionalism). Timonina used Cairney (2020) for a list of mainstream theories and concepts, which are also summarised on Cairney’s blog. Cairney performed a further inclusion check while analysing each article, referring some back to double check for exclusion. Cairney and Stephan double-screened 37 (and excluded 33) borderline cases during the final eligibility phase (using full-text analysis) (see Table 1 and Figure 1). Third, we used snowballing to make sure that we explain key reference points in the field (e.g. Hajer, Dryzek, Ostrom).

**Table 1.** Search results 2022.

Database	Search results	Duplicates	No access	Excluded	Included
Web of Science	2,137	657	150	1,271	59
Scopus	2,019	1,009	178	812	20
Proquest	2,182	493	60	1,600	29
Grand total	6,338	2,159	388	3,683	108

Routine data collection. We coded the following aspects of each article (in Excel):

- *Country/ region of study*. Compared to our other reviews, there is less concentration in a small number of Western countries, partly because of the global focus, and because Western researchers seem more likely to focus on other (often Global South) countries. 35 had a general international focus (plus 3 focusing generally on African states or cities and 2 on the EU). A total of 54 countries were named as part of individual or comparative studies: 19 focused (solely or comparatively) on the US, 9 Australia, 8 Canada, India, 7 Brazil, 6 South Africa, 5 UK, 4 China, Colombia, Germany, Guatemala, Sweden, Vietnam, 3 Nepal, 2 Cambodia, Cameroon, Ecuador, Guatemala, Indonesia, Kenya, Papua New Guinea, Poland, Peru, Singapore, Thailand, 1 Bangladesh, Belgium, Burkina Faso, Czech Republic, Denmark, Estonia, Fiji, France, Greece, Honduras, Italy, Jamaica, Japan, Kazakhstan, Korea, Lao PDR, Mali, Mekong/ East Asia, Myanmar, Niger, Netherlands, Nicaragua, Norway, Pakistan, Saudi Arabia, Spain, Sri Lanka, Trinidad and Tobago, Uganda.
- *Country of author affiliation*. Here, we find a greater concentration in Western countries (albeit less so than in our other reviews). Of the 108 authorial teams, 34 included a UK based author, 29 US, 17 Australia, 15 Canada, Netherlands, 14 Sweden, 12 Germany, 4 Denmark, Norway, Finland, 3 India, Italy, South Africa, Switzerland, Trinidad and Tobago, 2 Brazil, France, Indonesia, Kenya, Poland, Portugal, Singapore, and 1 Austria, Benin, Bulgaria, Cambodia, Columbia, Czech Republic, Ghana, Greece, LAO PDR, Mexico, New Zealand, Spain, Thailand, Uganda, Ukraine.
- *Policy or case study issue*. 74 (68%) focused on mitigating climate change, 23 (21%) focused on adaptation, and 11 (10%) on both. 55 (51%) studied climate change and sustainability in general, while 23 (21%) focused on energy, 11 (10%) forestry, 10 (9%) flood and other vulnerability to extreme events, and 6 (6%) agriculture or rural affairs (other topics included urban planning and migration).
- *Research methods*. 63 (58%) employ primarily qualitative methods (listed generally as qualitative, or focusing on discourse analysis, qualitative interviews, documentary analysis, and/or ethnography), 26 (24%) are literature reviews, 11 (10%) are quantitative (surveys), 5 (5%) are quantitative and qualitative (e.g. media coding), 3 (3%) involve policy analysis (including modelling or evaluation).
- *Article or book type*. 107 (99%) were journal articles (1 book chapter), and all were research texts (zero commentary articles).

We also gathered information in relation to three questions ( Cairney & Kippin, 2022: 7):

1. How do the authors (or their subjects) define concepts such as climate justice, equity, or just transition? (summarised in ‘The competition to define climate justice’)
2. What, if any, are their policy recommendations? (summarised in ‘What changes to policy or policymaking do climate researchers and policymakers seek?’)
3. On what policy concepts and theories do they draw (and cite)? We find that most texts make fleeting reference to policy theories (excepting 10+ references to Elinor Ostrom, usually while discussing socio-ecological systems) or concepts (excepting a broad focus on framing or governance in around 7 cases each). In comparison, at least 16 cite Hajer, 12 Dryzek, and 5 Fischer, and more cite an environmental policy-specific literature (such as by Sovacool and colleagues).

*Data analysis, aggregation, and presentation*. One author (Cairney) used an inductive qualitative approach to analyse each text, generate themes (Results), and relate them to policy theory insights (Discussion). As Cairney and Kippin (2022) note, ‘the rules associated with this method are less prescriptive than with its quantitative equivalent, suggesting that we (a) describe each key judgement, and (b) foster respect for each author’s methods and aims’ (see Sandelowski & Barroso, 2007: xv). The 20,000 word limit allows us to perform the latter. In a separate Word document, Cairney produced 3–400 word summaries of each text’s story (including research aims and question, approach, findings). Cairney condensed these summaries to produce thematic findings from the studies as a whole, and used a sub-set of relatively theory-informed texts to describe the results of engagement with policy concepts. We did not perform any quantitative tests to assess risk of bias. We present a narrative systematic review (rather than qualitative coding aided by tests for inter-coder reliability). We use general descriptions of ‘most’ or ‘few’ texts to aid that narrative, not signal precise proportions of the 108 included texts.

The complete search protocol, PRISMA checklist, and bibliography of the 108 included texts is stored on the OSF ( https://osf.io/f763m/) ( Cairney & Timonina, 2022).

## Results

### The policymaking context: IPCC, UNFCCC, and contested justice

There are three contextual reference points for most of the included texts. Combined, they suggest that there has been minimal but growing attention to climate justice, prompting greater attention to the contested definition and application of this concept.

***The IPCC provides an authoritative account of the scientific evidence on climate change, but how does it address climate justice?***

Few included studies centre the IPCC, but it still represents a focal point for wider political attention to a scientific consensus on the size and urgency of the climate change problem:

‘Human-induced climate change, including more frequent and intense extreme events, has caused widespread adverse impacts and related losses and damages to nature and people … The rise in weather and climate extremes has led to some irreversible impacts as natural and human systems are pushed beyond their ability to adapt’ ( IPCC, 2022: 11).

For example, the UNFCCC (2022a) describes the IPCC’s reports as ‘the most credible sources of information on climate change’. These testimonials are important in relation to two connected issues. First, there are inevitable ‘epistemic uncertainties’ in relation to predicting climate change and the likely impact of policy ( Hinrichs-Krapels & Biswas, 2023). Second, the exploitation of uncertainty is a regular feature of partisan debates and climate denial. Oreskes and Conway (2010: 2–7) describe the IPCC as ‘the world’s leading authority on climate issues’, to: describe its appeal to the majority of professionally recognised scientists; and, reject climate sceptic claims based on their support among a ‘handful’ of scientists, bolstered by ‘think tanks and private corporations’, sowing doubt regarding the ‘reality of global warming’. Similarly, Harris (2021) highlights the high scientific consensus behind IPCC reports, supported by its ‘rigorous selection process’, international coverage (‘721 scientists from 90 countries’) and quality assurance processes (see also Guay, 1999).

The IPCC’s role in relation to climate justice is less clear. First, it did not pay much attention to the concept until its third report (2001) when ‘crosscutting issues such as equity, discount rates, and decision making frameworks’ became an explicit part of its remit ( Mayda, 2000: 249). Second, previous attempts to feed-in cost-benefit-analysis to the IPCC highlighted problematic treatments of equity. For example, Azar (1999: 250) relates (a) calculations of damage in the Global South caused by high emissions in the North, to (b) early studies to inform IPCC work which placed a much higher ‘value of a statistical life (VOSL)’ to populations in the ‘industrialised world’ than in ‘low-income countries’ (see also Scovronick *et al*., 2020; Tol, 2001: 71). Third, an appeal to high scientific authority can have negative consequences, such as to depoliticise debates or marginalise other sources of relevant knowledge. There are signs of a shift in IPCC focus towards incorporating these concerns:

‘This report recognises the value of diverse forms of knowledge such as scientific, as well as Indigenous knowledge and local knowledge in understanding and evaluating climate adaptation processes and actions to reduce risks from human-induced climate change. AR6 [the 6 ^th^ IPCC report] highlights adaptation solutions which are effective, feasible, and conform to principles of justice’ ( IPCC, 2022: 9).
‘Maladaptation’ to climate change ‘affects marginalised and vulnerable groups adversely (e.g., Indigenous Peoples, ethnic minorities, low-income households, informal settlements), reinforcing and entrenching existing inequities’ (2022: 29)
‘Inclusive governance that prioritises equity and justice in adaptation planning and implementation leads to more effective and sustainable adaptation outcomes’ (2022: 30).

***The UNFCC provides an authoritative venue to discuss policy change, but what role does it play in climate justice?***

The UNFCCC was established in 1992 and has coordinated annual meetings of COP (the Conference of the Parties, or ‘supreme decision-making body’ of the UNFCCC) from 1995 ( UNFCCC, 2022b). Included articles tend to describe a small number of significant COP meetings – such as in Kyoto (COP 3, 1997), Copenhagen (COP15, 2009), and Paris (COP21, 2015) - in relation to their ability to produce international agreements on increasingly ambitious targets to mitigate climate change. They then describe less significant progress towards binding agreements to produce more equitable outcomes.

*Kyoto (COP-3)*. Rosen (2015: 30) argues that the Kyoto Protocol was a failure because it ‘stacked the deck against success in mitigating climate change’ (2015: 31). Problems included: key detractors (the US did not ratify; Australia withdrew); insufficient compliance with measures to produce modest GHG reductions (countries avoiding emission reduction targets included ‘China, India, and the rest of the developing world’); and ‘an inefficient climate regime complex’ consisting of many different venues and commitments (2015: 35–40). The failed design of Kyoto includes: (1) a short time-frame for action (5 years) after a delay (10 years), which encouraged countries to pick the ‘low hanging fruit’ (there was too little time to consider more fundamental reforms), (2) no promotion of innovation (it was too easy to meet targets with incremental changes), (3) the use of ‘net emissions’, which was too easy to game, and (4) the setting of a precedent for ineffective treaties (2015: 40). The process did little to foster procedural justice: smaller emitters had ‘little control over either negotiations or outcomes that will disproportionately affect them’, while women and marginalized groups (including Indigenous groups) ‘struggled for access, recognition, and representation’ and ‘have often been ignored’ ( Rosen, 2015: 34).

*Copenhagen (COP-15)*. Climate justice was a feature of COP15 negotiations, accompanied by public and media discourse that exposed contestation between climate activists versus skeptics. Audet (2013: 369) identifies contested definitions among different ‘bargaining coalitions’ (countries aligned via strategy or aims) at COP meetings, producing competing stories regarding climate justice:

‘The conflict discourse articulates the North–South duality over issues of historical responsibility for climate change. The transition discourse points to solving the problem of sharing the cost of mitigating climate change through a process of global low-carbon growth. The vulnerability discourse focuses on the urgency of ambitious actions by all parties’.

Wahlström *et al*. (2013) find minimal evidence of wider awareness of these ideas. They conducted surveys to see who was attending the climate demonstrations ‘organized by a coalition of environmental, religious, political, trade union, and solidarity organizations, embracing 538 organizations from 67 countries’ (and ranging from 15,000–100,000 people) and (2) what they believe should happen (2013: 4–5). They probed the extent to which a ‘climate justice’ frame is apparent as a ‘master frame’ to provide a bridge between many actors. They find that around half of those surveyed saw themselves as part of a ‘global justice movement’ (GJM) but with a tendency to discuss individualistic solutions. A more state interventionist form of ‘climate justice’ was not common, even though it was pushed by organisers (2013: 24).

Liang *et al*. (2014) analyse the US, Canada, and China TV coverage of Copenhagen and find minimal evidence that each country’s government intended to take ‘causal responsibility’ (admit fault) *and* ‘treatment responsibility’ (address the problem in the future). Rather, Canada takes responsibility for harm but not international leadership. The US seeks to share responsibility for harm with other developed countries, while blaming countries like China for limited international progress. China places the blame for harm on countries like the US (2014: 267–8).

Van Eck and Feindt (2022: 194) describe Copenhagen as ‘a watershed moment in the international climate change discourse, reinforcing controversy and polarization between climate sceptics and climate activists’. They analyse ‘the various storylines in the blog discourses of climate sceptics and climate activists between 2009–2015’ in the UK (following ‘climategate’, when ‘hacked emails by climate scientists provided climate sceptics with the opportunity to question the integrity of climate science’). Five climate activist storylines were ‘action’, ‘disaster strikes’, ‘potential catastrophe’, ‘opportunity’, and ‘social justice’ to describe (1) ‘inequitable vulnerabilities’ to climate change, (2) the countries that face the worst problems have caused the least, and (3) the need for ‘community voice’ in policymaking. Climate sceptic storylines include ‘hoax’, ‘no scientific evidence’, ‘climate sceptical science’, and relate ‘injustice’ to the idea of (1) climate change as a scam to protect the advantages of developed countries, or (2) ‘how environmental campaigns and protests were unethical and climate change policies were too costly’ (2022: 199–201).

*Paris (COP-21)*. The Paris Agreement may be era-defining in relation to the aim of:

‘Holding the increase in the global average temperature to well below 2°C above pre-industrial levels and to pursue efforts to limit the temperature increase to 1.5°C above pre-industrial levels, recognising that this would significantly reduce the risks and impacts of climate change’ ( UN, 2015: 3).

However, it provides a lacklustre and non-committal account of justice: ‘noting the importance for some of the concept of “climate justice”, when taking action to address climate change’ ( UN, 2015: 2). There is more reference to ‘equity’ (and ‘efforts to eradicate poverty’), including ‘the principle of equity and common but differentiated responsibilities and respective capabilities, in the light of different national circumstances’ (2015: 2), and acknowledging that Parties should respect their ‘obligations on human rights, the right to health, the rights of indigenous peoples, local communities, migrants, children, persons with disabilities and people in vulnerable situations and the right to development, as well as gender equality, empowerment of women and intergenerational equity’ (2015: 3). However, there is a general reluctance to oblige countries to act, in favour of vague agreement on a less-binding plan ( Dimitrov, 2016: 6–7; Falkner, 2016; Livingston & Rummukainen, 2020). The agreement also depoliticises key aspects by asking the IPCC to make sense of its aims ( King *et al*., 2017).

In that context, Sokołowski and Heffron’s (2022: 4) list of indicators of ‘policy failure’ includes the description of goals ‘in an ambiguous, constrained, or contradictory manner, with no measurable criteria, or timeline’. Further, Mills-Novoa and Liverman (2019: 1–2) identify confusion about how ‘the 10 top greenhouse gas emitters and the founding countries of the Climate Vulnerable Forum (CVF)’ will deliver on Nationally Determined Contributions (NDCs; their GHG reduction plans and requests for support). They identify competing discourses from NDC reports, including: who they blame or hold responsible for emissions; developing countries pledging to act only if developed countries do so, or tying pledged action to financial support; and, more or less support for carbon markets as a key means of GHG reduction (2019: 3–10). They also identify ‘silences in the NDCs’, including limited reference to scientific evidence, the ‘lack of any discussion of embodied emissions or the emissions created through the production, processing, or transport of goods’, and sparing references to climate justice in relation to gender or Indigenous groups (2019: 10).

***‘Climate justice’ and ‘just transition’ are essential but contested aims***

Climate justice is ambiguous and contested ( Newell *et al*., 2021). It is straightforward to describe climate justice vaguely as the need to produce policies to address climate change while ensuring that policy processes and outcomes are respectful and fair. The IPCC (2022: 9) also identifies broad categories, in which fairness relates to outcomes (who gets what?), processes (who decides?), and respect (whose knowledge and experiences count?). Further, we would expect most research to compare hopes for climate justice with actual systematic injustice, in which climate change is: ‘having the most severe effects on those with the least responsibility for causing it, and who, at the same time, are often excluded from decision-making processes regarding responses to the problem’ ( Newell *et al*., 2021: 2). This problem relates to inequalities of power, such as in relation to current and future generations (intergenerational injustice), more or less powerful countries, and the gendered and racialised impacts of climate change and climate policy (2021: 6; Mattar *et al*., 2021: 1307; Terry, 2009).

Turning these abstract categories and aims into concrete definitions and objectives is problematic because there is no ‘universalist philosophy of justice’, and its practical meaning varies in relation to factors such as geography, and if the narrative comes from academics or activists ( Newell *et al*., 2021: 2). Further, academic descriptions of fairness differ in energy, environmental, and climate studies ( Heffron & McCauley, 2018: 74; Jenkins, 2018).

### The competition to define climate justice: fostering social justice approaches

Many included texts acknowledge this contestation to define what justice and equity mean, and they generate a wide range of context-specific applications (e.g. Audet, 2013; Schmidt & Schäfer, 2015). There is some variation according to a discussion of *mitigation,* focusing more on global issues such as who is disproportionately responsible for the problem (and solution) or most likely to suffer the effects, or *adaptation*, focusing more on specific contexts, including the relationship between national and subnational policies and the inclusion of local communities or citizens in policymaking. These differences also vary according to sector or issue, which can include schemes to transition from fossil fuel consumption to renewable energy (largely classified as mitigation), flood prevention or reaction policies, and agricultural schemes in relation to flood or drought.

Nevertheless, there are strong overlaps between definitions, in which most cohere around a broad social justice account which they contrast with a neoliberal approach (the latter clearly has powerful support, but not among academics publishing articles on climate justice). Most social justice definitions provide variations on the same theme of recognitional, procedural, and distributional justice:

- ‘Distributional justice can be defined as fairness in the distribution of benefits and harms of decisions and actions to different groups across space’
- ‘Procedural justice refers to the level of participation and inclusiveness of decision making and the quality of governance processes’
- ‘Recognitional justice refers to the acknowledgement of and respect for pre-existing governance arrangements as well as the distinct rights, worldviews, knowledge, needs, livelihoods, histories and cultures of different groups in decisions’

(Quotations taken from Bennett *et al*., 2019: 4–5; see also Burley *et al*., 2012; McLaren & Corry, 2021; Nielsen, 2014; Pellegrini-Masini *et al*., 2020; Sakellari, 2022: 69; Valdivia & Balcell, 2022: 6; see also Carter, 2007: 62; Carter, 2018: 63 on ‘social justice’).

***Distributive justice: a fair allocation of costs and benefits in relation to mitigating, adapting to, or suffering the effects of, climate change***

Articles describe the importance of equitable allocations of benefits and burdens, but what is the specific aim or means to do so? For example, the UNFCCC adopted the ‘principle of common but differentiated responsibilities’ so that ‘developed nations … should take the lead in combating climate change and its impacts’ ( McGee & Taplin, 2009: 220; Van Eck & Feindt, 2022; see also Severson & Coleman, 2015: 1282 on ‘poor island countries’). Further, there is a frequent focus on Global North countries compensating or supporting Global South countries:

‘it is the poorest countries of the global South that will be most adversely affected, either because their already precarious ability to sustain human life will be tipped over the edge, or because, lacking the resources that make no less adversely affected countries relatively resilient, they are likely to suffer severe social, economic and possibly political dislocation’ ( Rootes *et al*., 2012: 677; see also Fløttum and Gjerstad, 2013: 61).

However, Okereke (2010: 467, Table 1) summarises many ways to translate broad values into a rule for all countries to follow in relation to GHGs (focused more or less on justice):

- ‘Equal per capita’ or ‘Sovereign’ equality. Each *person* or *country* has an equal share of allowable emissions or the same ‘entitlement and burden’.
- ‘Status quo/grandfathering’ versus ‘Polluter pays/historic responsibility’. Each country *maintains* or *recalculates* entitlements in relation to their historic or current emissions (to maintain an advantage versus to pay - and compensate others - for past benefits).
- ‘Basic need’. ‘Allocate rights to survival emissions and share remaining burden to benefit the least well off’.
- ‘Property rights’. ‘Create tradable permits to achieve lowers net world cost for abatement’.
- ‘Mutual advantage’. ‘Allocate benefits and burden’ to ensure a ‘positive net benefit for all’.
- ‘Kantian allocation rule’. Countries accept the rules/ limits for themselves that they would seek for others (in comparable circumstances) (see also Häyhä *et al*., 2016; Huitema *et al*., 2016: 7; Kleinen-von Königslöw *et al*., 2019: 518–21; Pillai & Dubash, 2021; Stoddart *et al*., 2012; Wardekker *et al*., 2009: 518; compare with Cazorla & Toman, 2000; Rose *et al*., 1998).

More specific domestic examples include the need to address low-income, poverty, or socio-economic inequality in relation to:

- *Forestry*. Redistribute the benefits of REDD+ (an economic incentive scheme to protect forests) to reduce poverty in countries including Brazil, Cameroon, Ecuador, Indonesia, Nepal, Papua New Guinea, Peru, and Vietnam ( Brockhaus *et al*., 2014: 29; da Conceição *et al*., 2015: 247; Mbatu, 2020: 1–2; see also Sarkki *et al*., 2017: 32 on ‘fragile treeline areas’ in Europe).
- *Energy poverty*. Address energy or fuel poverty ( Baker, 2016: 795 on South Africa; Butler *et al*., 2018 on the UK) or unequal access to fuel ( Knox-Hayes *et al*., 2013, comparing Brazil, China, Germany, India, Kazakhstan, Japan, Papua New Guinea, Saudi Arabia, Singapore, and the United States). Compensate low-income families in Australia for ‘climate mitigation policies’ such as carbon taxes ( Copland, 2020: 625), and leave ‘no person or place behind’ when delivering a Green Deal in the EU ( Skjærseth, 2021: 26).
- *Events such as floods or drought*. Address a loss of income related to climate impacts on the Mekong river ( Gerlak & Schmeier, 2014: 367). Address South Africa’s ‘significant socio-economic inequalities’ to reduce the extent to which they exacerbate the unequal ‘loss of life, health and property’ associated with adaptation ( Fløttum & Gjerstad, 2013: 61), such as in relation to ‘informal dwellers’ facing urban flooding ( Hetz & Burns, 2014: 887). Resilience to floods (in the Netherlands) is ‘diminished for deprived households, single parent households, elderly or ethnic minorities’ ( Kaufmann *et al*., 2018: 326). There is insufficient focus on who can choose to live in flood risk areas ( Harries & Penning-Rowsell, 2011: 196 on the UK).
- *Agriculture*. Address farmer poverty related to climate impacts ( Lebel *et al*., 2018 on Lao PDR and Cambodia).
- *Sustainable development and adaptation*. Avoid benefiting disproportionately ‘the middle class and elites rather than the poorer social strata’ ( Leck & Simon, 2018: 4–5 on South Africa).
- *Energy transitions*. Recognise the economic losers of phasing out fossil fuel extraction ( Lehotský *et al*., 2019: 774 on the Czech Republic).
- *Intersecting forms of inequalities*. Address a tendency for climate change to exacerbate existing inequalities, in relation to the ‘social determinants of health’ ( Bowen *et al*., 2012), or relative ‘vulnerability’ or ‘inequalities in the resources available to adapt’ ( Huitema *et al*., 2016: 7; Popke *et al*., 2016: 79).

A small proportion of research focuses on arguments counter to this broad consensus. Zuk and Szulecki (2020; x) describe far right populist arguments that relate energy justice to protecting national interests against foreign and EU interference (in relation to coal in the Polish economy). These arguments can include conspiracy theories about ‘leftists’ or Germans seeking to take over Polish mines (see also Lockwood, 2018; compare with Van Eck and Feindt, 2022: 194 on climate sceptic social media in the UK, and Schmidt & Schäfer, 2015: 535 on contested notions of climate justice in German, Indian and US media).

***Procedural justice: a fair process to make choices about how to address climate change***

Procedural fairness can describe:

- A minimal focus on a formal opportunity for widespread consultation.
- A maximal focus on institutionalising the principle (such as in the South Africa constitution or Kenyan Climate Change Act 2016 - Colenbrander, 2019; Naeku, 2020) *and* creating the conditions for meaningful participation, such as by addressing imbalances in power or the resources to participate effectively ( Bertana, 2020; Brink & Wamsler, 2018: 92; Crosweller & Tschakert, 2021: 3; Muncie, 2021; Sovacool *et al*., 2020: 16).

Malloy and Ashcraft’s (2020: 3) review of ‘conceptualizations of injustice’ in adaptation studies exhorts policymakers to: ensure ‘socially vulnerable populations gain the political power to shape adaptation decisions’, frame problems to ‘recognize adaptation needs of vulnerable populations’, ‘recognize and account for the desired needs and wants of traditionally marginalized groups’, and ‘substantively address the structural conditions that produce participatory inequality, such as poverty, exclusion, or the role of culture’.

The value of procedural justice can be self-evident (the right thing to do; an end in itself) or instrumental (to increase support for policy change). For example, ‘processes perceived to be inclusive, meaningful, and fair increase local support for wind energy’ ( Walker *et al*., 2018: 678) or ‘climate-friendly policies’ ( Pellegrini-Masini *et al*., 2020: 5–6), while procedural unfairness undermines the sense that a company has a ‘social license’ to operate ( Cotton *et al*., 2014: 433 on fracking in England; Songsore & Buzzelli, 2016 on renewables development in Canada).

Copland (2020: 623 on Australia) compares high general government commitment to tackle climate change to effective opposition to specific policy changes (such as the emissions trading scheme), relating to ‘anti-politics’ protest or action (in other words, fuelled by disenchantment with politics or distrust of politicians – Beveridge and Featherstone, 2021). Copland (2020: 624) argues that policymakers need to understand legitimate concerns based on opposition to top-down state action without sufficient stakeholder or citizen participation. Similarly, Anderson and Schirmer (2015:7 62 on Australia) warn of high and effective opposition to new renewables developments when people feel an attachment to ‘place’ and think that developers/ governments oversee tokenistic participatory processes. Walker *et al*. (2018: 670 on onshore wind energy in Canada) find that a party whose support and election comes largely from urban areas can get punished in rural areas if there is a successful campaign to politicise the urban/rural divide and pursue the narrative that urban parties push the costs to rural areas. Gray and Bernell (2020: 7) describe the lack of procedural justice which undermined respect for an otherwise well-supported Oregon Clean Electricity and Coal Transition Plan.

***Recognitional justice: ensuring that people are recognised as a distinct social group with the right to be heard***

Recognitional justice overlaps with procedural justice, but focusing on the injustices that arise when social groups are unable to ‘affirm a specific group’ because they are ‘misrecognised’: ‘the result of intentionally created and/or maintained institutionalised structure that depreciate vulnerable groups’ ( da Costa Silva, 2011: 448). Marginalisation can relate to:

- The intersection of gender, race, age, socio-economic status, and/or sexuality (2021: 448; Acosta *et al*., 2019; Alverio *et al*., 2021: 515; Burch *et al*., 2019: 5; Di Gregorio *et al*., 2013; Holmgren & Arora-Jonsson, 2015; Jerneck, 2018: 403; Romsdahl, 2020: 159; Mangat *et al*., 2018: 200; Zannakis, 2015: 219). Godden *et al*. (2020: 595) seek to challenge discrimination and ensure fair participation to ‘increase women’s access to justice and strengthen their political leadership and participation in decision-making processes’ and build ‘the capacity of the most marginalised, indigenous, migrant, and poor women on their rights over land, resources, decent work, peace, and security’.
- Specific groups in varying contexts, including ‘frontline community stakeholders’ in California ( Basseches *et al*., 2021: 41; Fernandez-Bou *et al*., 2021: 1), or people whose ‘livelihoods … are invested’ in an ‘at-risk location’ subject to climate-related relocation ( Alverio *et al*., 2021: 515)
- The marginalisation of civil society groups in authoritarian states (e.g. Smits, 2017: 79 on Vietnam and Thailand; Teo & Amir, 2021: 203 on Singapore).
- The silence on such matters in existing studies ( Liebenguth, 2020: 193–95 on environmental security discourse), key organisations ( Mikulewicz & Taylor, 2020 on the World Bank), or climate change strategies ( Mills-Novoa & Liverman, 2019: 10; Simon & Leck, 2015: 113).
- The dominance of a Western lens at the expense of perspectives from ‘traditional and Indigenous approaches’ ( Werners *et al*., 2021: 173–4), or ‘patriarchal systems that privilege the voices and power of white men over women or people who are not white’ ( Sovacool *et al*., 2020: 9; see also Sovacool *et al*., 2017).

***Other narratives of justice: human rights, restorative, cosmopolitan frames***

Public policy consists of norms, rules, and principles that ‘offer legitimacy for government to take measures, provide compensation, use regulatory or economic instruments, and protect rights’ in the ‘public interest’, but with human rights and ‘public interest’ contested in principle and practice ( Huitema *et al*., 2016: 5). This debate includes mixed views about which vehicles for climate justice are the most appropriate and effective. Many texts emphasise the role for ‘human rights’ frames, including the right to breathe clean air and have access to unpolluted water, or a more general right to wellbeing. This approach may empower ‘people and communities to live in a way that respects nature and cultural diversity and guarantees human rights and the rights of indigenous peoples’ ( Partelow *et al*., 2020: 10).

Jodoin *et al*. (2020: 169) explore the idea that litigation drawing on ‘human rights norms and arguments’ could help to bolster collective action without undermining existing approaches. Aims include to increase attention to climate justice, foster ‘alliances between climate justice activists and other social movements’, ‘mobilize citizens from disadvantaged segments of the population’, challenge the tendency to treat environmental problems technocratically (favouring climate scientists and economists) and challenge academic scepticism about using courts for transformative social change (2020: 168–9). However, campaigners also face the ‘delicate task of seeking to position their claims in a way that seeks to engender transformative social or political change, while also being somewhat consistent with the ideas that prevail in a given culture, community, or institution’ (2020: 176). Their case study is of ‘a petition submitted by Inuit communities in the arctic on the human rights violations caused by climate change before the Inter-American Commission of Human Rights in 2005’ (2020: 168). They find support for the human rights claim among ‘climate lawyers and activists in the global climate justice movement’ (2020: 192). However, it enjoyed ‘little resonance among policymakers in the United States and Canada’, who saw it as an economic threat, *and* among ‘the Inuit communities on whose behalf it was filed’ since ‘Western confrontational activism’ is ‘in many respects incongruent with existing Inuit cultural, social, and legal norms and practices’ (2020: 188–92).

Other concepts help to bolster social justice definitions in energy justice research. Sokołowski and Heffron (2022: 4) describe five elements: ‘procedural, distributive, restorative, recognition, and cosmopolitan’. Restorative describes rectifying injustices (such as fossil fuel companies compensating others for the damage they caused), while cosmopolitan describes the need to consider ‘cross-border effects’ of energy production and use since ‘we are all citizens of the same world’ (2022: 5). Pellegrini-Masini *et al*.’s (2020) review finds adherence to recognition, procedural, and distributional justice, as well as auxiliary principles to: ensure that people have access to affordable and sustainable energy, foster equity among current and future generations, deliver ‘due process’ (including transparent policymaking and protecting human rights), and take an intersectional approach to recognise how social groups are affected (2020: 2). Similarly, in climate media research, Kleinen-von Königslöw *et al*. (2019: 523) expand on the three principles to show how political actors make claims of legitimate action, in relation to the ability of ‘all affected citizens’ to be represented or able to participate directly, a transparent and deliberative political process (with ‘no hidden agenda’), the ‘empowerment’ of citizens, the protection of human rights, and policy for the ‘common good’ rather than ‘just privileged elites’.

***Define climate justice to help explain climate injustice***

Defining climate justice is the first step to identifying a lack of attention to climate *injustice*, such as in academic research ( Burch *et al*., 2019: 5 describe ‘decades of neglect’), among many environmental NGOs ( Partelow *et al*., 2020: 1), and in policy texts ( Fløttum & Gjerstad, 2013: 63). In policy analysis, some identify the effects of a combination of all three injustices (e.g. Koch & Verholt, 2020 on the impact of financial incentive schemes on ‘smallholders’). Many show that a lack of ‘recognition’ is an essential but underexplored element, such as when it does not occur to policymakers to think about specific social groups.

Jerneck’s (2018: 403–4; 412) analysis of agricultural adaptation in Sub-Saharan African states finds a lack of ‘gender sensitive’ strategies and a tendency to take for granted the ‘gendered divisions of rights and responsibilities’ that are ‘expressed in power asymmetries in access to land, labour, and leisure time’. 

Sovacool’s (2021: 1–2) review of energy transitions shows that a focus on justice shows what can go wrong during mitigation. It is tempting to assume that low-carbon energy production is more ‘equitable, egalitarian, and just than their fossil-fueled or carbon-intensive counterparts’. However, transition presents opportunities to perpetuate inequalities, fuelled by the perpetuation of ‘ *entrenchment’* (elites using new renewable projects to boost their own income and power; the further marginalisation of women in new projects); ‘ *encroachment’* (e.g. biofuel companies taking over crops and devastating forests); ‘ *exclusion’* (energy companies producing new projects while excluding local communities); and, ‘ *enclosure’* (e.g. companies forcing communities to relocate from their land) (2021: 7). Sovacool’s review finds frequent examples of climate injustices among:

‘no less than 61 different vulnerable groups of indigenous peoples, aboriginal collectives, or ethnic minorities negatively impacted by climate efforts, some of them under threat via multiple processes or mitigation options at once. Moreover, the impacts differentially cut across: scales (spanning local-community divides or urban–rural locations or even implicating global actors such as unions, financers, or investors); temporality (present vs. future generations); and recognition (affecting very specific and highly vulnerable groups including child prostitutes, slaves, smallholder farmers, coastal property owners, etc.)’ (2021: 13; see also Sovacool & Linnér, 2016)

### The competition to define climate justice: Opposing neoliberal approaches

Our interpretation is that the most common explanation for the perpetuation of injustice (in the included texts) is that a UNFCCC-led focus on climate change has not been accompanied by a transformation towards a social justice policy paradigm. Rather, social justice is tacked onto a globally dominant neoliberal approach to policy, which often relies on weak forms of ‘ecological modernisation’. Broadly speaking, the latter is a rejection of radical reforms to state and market, in favour of ‘greening’ by modifying the existing economic and institutional structures ( Carter, 2018: 232). In general, neoliberalism privileges economic growth and free trade and favours market mechanisms (or individual responsibility) over major state intervention. Variations of this argument include:

‘A neoliberal capitalist discourse dominates global affairs, with devastating effects for ecological integrity and social justice … Seeking endless economic growth, free markets and small government, it has promoted unconstrained exploitation of people and planet, leading to ecological destruction and human misery … [contributing] to inequality, concentration of wealth, financial instability, social injustice, destruction of nature and other commons, and intrusion of market logic into community life’ ( Riedy, 2020: 103).
‘Feminist activists argue that the gendered injustices of climate change are caused by globalisation, fundamentalisms, militarism, and patriarchy – a neoliberal development model of power and control that exploits women and the environment for global corporate profit ( Godden *et al*., 2020: 594).
‘Civic environmentalism identifies economic growth, industrialism and capitalism as the root causes of environmental degradation. To manage them, the discourse focuses on issues of social justice, decentralization of power and especially a concern for marginalized groups’ ( Zannakis, 2015: 219).

Further, supporters of neoliberal approaches seek to ‘depoliticise’ policy and policymaking by (1) describing low state intervention or market mechanisms as the natural or default option, and (2) cloaking climate change policy in a technocratic language, thus emphasising the role of scientific or other experts at the expense of more participatory or deliberative processes:

‘Neoliberal environmentalism … relies on technical “experts” to function and legitimize apolitical interventions. Environmental neoliberal interventions are often depicted as common sense, objective or neutral through a process of depoliticization, or “to remove issues from political contention”, as opposed to value-laden and normative, political, issues due to considerations of equity and justice. “Expert knowledge,” then, becomes a way to empower market actors and others while marginalizing locals and context-specific concerns’ ( Johnson, 2021: 4)

The World Bank is a common focus of criticism since it fosters ‘neoliberal approaches to climate change policy’ through (1) its preferred discourse of ‘resilience’, to treat climate change ‘as an external threat to an otherwise seamless narrative of African advancement’, and (2) ‘aggressively apolitical decision-making rooted in techno-managerialism and characterised by furthering technological and institutional solutions to what are projected as technical problems’ (which obscures who wins and loses, and downplays the need to discuss equity), while (3) using its control of funding to set the agenda for recipients (‘$19bn of loans, grants and investment’) under the World Bank Africa Climate Business Plan ( Mikulewicz & Taylor, 2020: 626–7)

***Contrasting the right and wrong approaches***

Although it takes several forms, there is a consistent focus on the contrast between a favoured social justice approach (which informs CE) and an unfavoured neoliberal approach (informing weak EM). Table 2 sums up the broad contrast from the perspective of climate justice researchers.

**Table 2.** Neoliberal versus social justice environmentalism.

	Neoliberal informed	Social Justice informed
The role of state and market	Harness markets to produce efficient solutions	Use state intervention to produce equitable solutions
Favoured policy instruments	Economic incentives, such as via carbon markets or rewards for environmental protection.	Regulations on individual and business behaviour, measures to redistribute costs and benefits (or income), biodiversity schemes.
The primacy of economic growth	Seek win-win-win solutions where climate policies boost economic, environmental, and social aims	Engage with the inevitable trade-offs between economic growth, environmental sustainability, and social concerns.
The role of technological innovation	High emphasis on the ability of technological innovation to solve the climate crisis.	Technology has a valuable role in energy system transformation but should not be a seen as a panacea or reason to avoid debate.
The role of business/ corporations	Essential partners in the pursuit of policy and technological change.	Too-powerful actors who benefit from capitalism and halt policy change.
The role of participants and knowledge	Treats climate change as a problem amenable to technical solutions, favouring scientific and economic experts, engineers, and managerialism.	Treats climate change as an inescapably political problem, favouring stakeholder and citizen participation and deliberation on values (such as regarding social protection and redistribution). Respect non-Western/ scientific conceptions of knowledge.
The role of multi-level governance	Top-down and centralist.	Frequent focus on decentralised governance, to place value on local collaborative governance.

Source: adapted from Johnson (2021), Lebel *et al*. (2018), Nielsen (2014), Romsdahl (2020), Stephan & Paterson (2012), Teo & Amir (2021). See also Carter, 2018: 332–63 on policy instruments.

This broad contrast is subject to more nuanced discussion in each text, For example, Johnson (2021: 5) describes civic environmentalism as ‘a heterogeneous term’ to sum up:

‘concerns of environmental justice, ecological sustainability, equity, local knowledge systems and the inclusion of local stakeholder participation. This discourse is critical of EM’s mainstream discourse and its reliance on markets and technical experts to solve environmental problems … [questioning] the “win–win–win” storyline of ecological modernization, arguing that community-based conservation inherently involves trade-offs and highlights the disjuncture between ideals of poverty alleviation and actual practice. Furthermore, some civic environmentalists argue that community-based conservation is just a tool for the expansion of neoliberalism to further capital accumulation into rural areas’ (2021: 5–6; compare with ‘ecological justice’ in Zannakis, 2015).

Further, McGee and Taplin (2009) identify multiple texts in which the main contrast is between different *variants of EM*. ‘Weak’ EM is akin to the neoliberal approach: ‘economistic, technological, instrumental, technocratic, neocorporatist, national and unitary’, and weak in the sense that it is unlikely to promote ‘enduring ecologically sustainable transformations and outcomes across a range of issues and institutions’ (2009: 215–6, citing Christoff, 1996: 490). In contrast, ‘strong EM’ would ‘encourage an ecological, open, deliberative, communicative, international and diversified social structure’, while deliberative processes ‘would open up consideration of the normative assumptions of current development practices and potentially allow deep transformation of socio-economic systems away from current patterns of industrial modernity’ (2009: 215–6, citing Christoff, 1996: 496 and Dryzek, 2005: 173–174; compare with Carter, 2018: 232–7).

***The lack of a coherent and well understood radical alternative***

One unintended consequence, of such a strong focus on the wrong approach, is that few studies engage with the ambiguity of their own. Grist (2008: 783–6) identifies a lack of mutual understanding within social justice research, based on (1) underexplored epistemic differences in research (such as between positivist versus interpretive or postpositivist approaches) and (2) the sheltering of a broad range of approaches under the ‘radical’ umbrella.

Riedy (2020: 108) identifies a contrast between the (1) high certainty of researchers to identify the damaging impact of neoliberal paradigms, versus (2) ‘much uncertainty across alternative discourses’ about how to articulate and pursue a radical alternative. The unfulfilled aim is to produce a narrative of transformation that is acceptable to a large-enough coalition of actors with the power to take over institutions, practices, and policies in the same way as neoliberal actors have managed (2020: 100). There is a vague commitment to:

- Novelty, or ‘the need for a systemic orientation, a dialogical approach, new participatory and action-oriented approaches to knowledge generation, new forms of human consciousness, and genuine achievement of the ecological sustainability and social justice represented by the SDGs, without retaining commitment to their neoliberal baggage’
- A shift of policy aims from economic growth to ‘human dignity, prosperity and wellbeing for all’ (2020: 104–87).

However, there is also uncertainty or disagreement regarding (for example) the prospect for collaborative versus radical direct action, or more global or localised action. For example, multiple texts cite Bäckstrand and Lövbrand (2016: 519), who identify three different ‘Contending Climate Governance Discourses in the Post-Copenhagen Era’: (1) ecological modernization is conducive to ‘polycentric’ rather than top-down governance, (2) ‘green governmentality’ is the more international top-down and technocratic approach, and (3) ‘climate justice’ is associated increasingly with more radical ‘grass-root activism and protest’ to seek transformations from ‘global capitalism and neo-colonialism’ (2016: 528).

One exception comes from Godden *et al*. (2020: 603), who find that a more specific focus on challenging gendered injustice – via the ‘Feminist Fossil Fuel Free Future (5Fs)’ – is ‘highly effective in mobilising women, especially those from low-income and indigenous groups, to demand climate justice collectively’. Initiatives include ‘capacity building’ (including knowledge sharing and advocacy) to challenge discrimination, and advocate to ‘ensure international and regional laws, norms, standards, and practices reflect women’s human rights’, as well as ‘interrogate trade and investments rules and halt the growing power of corporations’ (2020: 595).

### What changes to policy or policymaking do climate researchers and policymakers seek?

We find a tendency to focus on (1) what authors *need to happen*, or require of political systems (profound changes to politics, policy, policymaking), without (2) relating that need to reasonable expectations of *what actually happens* ( Cairney *et al*., 2022a). Almost all of the articles that describe policy change highlight the need for transformations in relation to society (to change from unsustainable to sustainable behaviour), politics and policymaking (to foster recognitional and procedural justice), and policy (to reject neoliberal policy instruments). Then, they highlight a major gap between these requirements and reality, generally without using policy studies to help to explain the difference.

Most researchers use variants of this social justice approach to identify what is required. An exemplar is Bennett *et al*. (2019: 5):

‘Just transformations refers to radical shifts in social–ecological system configurations through forced, emergent or deliberate processes that produce balanced and beneficial outcomes for both social justice and environmental sustainability. Just transformation management consists of deliberate governance processes and actions taken to shift systems towards environmental sustainability and social justice outcomes in ways that account for recognitional, procedural and distributional concerns’ (2019: 5)

They describe the *need* for (1) transformational changes to politics and policy paradigms to produce higher levels of state intervention to secure environmental sustainability (‘radically transform the way we manage numerous marine, freshwater and terrestrial systems in rural and urban environments to promote environmental sustainability’) and (2) to address the unequal positive and negative consequences (given ‘a real danger that deliberate sustainability transformations will occur in an exclusionary manner or produce inequitable outcomes across time and space’) (2019: 1–2).

This focus on requirement is one of the most frequent themes in the literature, including:

***The need to transform systems of governance***

- Burch *et al*. (2019: 13) identify ‘the need to reshape governance systems at all scales within the Anthropocene’ since the current system is ‘wholly unprepared for the new challenges arising’ (see also Sarkki *et al*., 2017 on ‘synergistic goals for governance’).
- Leck and Simon (2018: 7) argue that ‘further adaptation initiatives will require innovative governance and partnering to enable step-changes in addressing the increasing stresses heralded by environmental change … thinking in transformative terms about the conditions and nature of change required in addressing climate change … tackling root causes of vulnerability, opening up opportunities for revision and replacement of existing unsustainable development trajectories and technological path dependencies, the successful negotiation of power relations, building empowerment, encouraging innovation, and protecting positive gains such as the inclusive modes of governance which have already been achieved’.

***The need to ‘mainstream’ environmentalism and justice across (and outside of) government (also known as policy integration)***

- McCauley (2015: 274) describes the need to include environmental groups in policymaking as part of a wider pursuit of ‘environmental policy integration’. This call for more integration is frequent ( Bowen *et al*., 2012; Burley *et al*., 2012: 587; Butler *et al*., 2018; Macintosh, 2013: 1050–1; Valdivia & Balcell, 2022: 1; see also Tanner *et al*., 2019).
- Smits (2017: 82) identifies (in Thailand and Vietnam) the need for ‘good coordination between parties within and outside the government if fragmentation is to be reduced’
- Mani *et al*. (2021) (on land degradation in South Africa) describe a need for faster reforms driven by more effective governance processes, to fulfil multiple aims of development, climate change mitigation and adaptation, and poverty reduction.

***The need to organise or collaborate to overcome neoliberalism***

- Brockhaus *et al*. (2014: 30) identifies the need for a social justice coalition to challenge the dominant neoliberal approach to REDD+. Mbatu (2020: 11 on Cameroon) finds some evidence of ‘stakeholder participation’, but also ‘superficial’ NGO engagement in which ‘virtually no national civil society organizations’ were represented.
- Stephan and Paterson (2012: 545) identify the need to: ‘deconstruct’ or problematise the ‘taken-for-granted routines and norms of carbon markets’, examine their history, and interrogate common practices and technologies (2012: 550–5).
- Fernandez-Bou *et al*. (2021: 1) argue that policymaking ‘must be grounded in collaboration with frontline community members and practitioners trained in working with vulnerable stakeholders’.

Few studies base these requirements on actual practices. A partial exception is da Costa Silva’s (2011: 446) pursuit of a ‘framework of environmental justice’ based on the established framework called ‘integrated water resource management (IWRM)’. Da Costa Silva (2011: 456–7) identifies (1) considerable evidence of decentralisation to municipal levels and greater participation among marginalised groups, albeit with (2) minimal evidence that the outcomes helped ‘to overcome significant social inequities’.

***The design of effective and equitable policy tools***

Policy design presents four multi-faceted dilemmas. First, policy and policymaking are not separable in practice, prompting policymakers to ask two questions at once: (1) which policy tools or instruments are appropriate, and (2) how should they be implemented?

Second, the answer to both questions is based on empirical questions (are these solutions technically and politically feasible?) and normative questions (are they the right things to do?). For example, Huitema *et al*. (2016) compare the alleged merits of approaches to governance. Top-down implementation by a single authority helps to set a clear direction, then monitor and evaluate performance, to hold people to account for failures to deliver. Bottom-up approaches decentralise that process, to give local policymakers and stakeholders more autonomy to design solutions, and accept ‘that goals are ambiguous and implementation “gaps” are the norm rather than the exception’ (2016: 5). The former may appeal to advocates who are sceptical about the weight that politicians put behind their stated ambitions. The latter is consistent with social justice models that stress high participation and deliberation.

Third, what happens when – as in multi-level systems, without the ability of one to direct all others – governments need to cooperate to co-produce a coherent collection of policy instruments? This issue raises the importance of ‘meta-governance’, or how to govern governance effectively (2016: 4).

Fourth, what are the relative advantages of different kinds of policy instrument? Huitema *et al*. (2016: 5) discuss three categories - redistributive (taxing some and giving the proceeds to others), distributive (providing goods and services to some social groups), and regulatory (setting and enforcing boundaries on individual and business behaviour) – and note the different political processes associated with each measure. Highly redistributive measures are often off-limits, distributive measures are appreciated only by some groups, and regulations are low cost to government but brook opposition by non-governmental actors.

Few studies engage fully with such dilemmas, but they contribute to different aspects. Häyhä *et al*. (2016: 60–1) provide an exemplar of describing a problem and leaving solutions to government: the ‘planetary boundaries framework’ identifies ‘an acceptable amount of human impact’ then invites policymakers to relate these limits to policy. Several criticise a reliance on quasi-market measures, where the state incentivises people and businesses to act environmentally ( Brockhaus *et al*., 2014; da Conceição *et al*., 2015, and Johnson, 2021 on REDD+; see also Grist, 2008: 796). Some seek innovation and a mix of (market-based and regulatory) instruments to influence business behaviour (e.g. Zanon & Verones, 2013: 343 on Italian cities). Several explore the extent to which public support for each instrument varies by country (or sector):

- Clayton (2018: 219) conducts a small US survey (162 residents) to identify public support for environmental policies. Responses highlight the importance of perceived fairness and suggest that ‘non-coercive’ policies are preferable (compared to a German survey which found higher support for tax and regulations since they applied to everyone and punished wrongful behaviour – 2018: 220).
- Stoddart *et al*. (2012: 39) analyse ‘open-ended questions completed by 1,227 members of nine different environmental organizations’ in Canada. They list what NGOs think governments should do, including ‘laws, regulation, enforcement’ at 28%, ‘education’ 15%, ‘punitive taxation, carbon tax’ 12%, tax incentives 10%, and public transport investment 10% (2012: 50; see also Bowen *et al*., 2012).
- Spandagos *et al*. (2022: 1) present a multi-country EU survey on public attitudes towards ‘sustainable energy innovations and policies’ (such as ‘electric vehicles, residential solar panels, energy-efficient home insulation, environmental taxes and incentives’). The distribution of costs and benefits affects support for environmental taxes, although they remain less popular than incentives ( Spandagos *et al*., 2022: 3 also cite multiple surveys of US, Canada, Norway, Germany, Australia, Greece, UK, Denmark, Czech Republic, Italy, France, Switzerland, Spain, Italy, Tunisia, Egypt, Jordan, Lebanon, Denmark, Germany, Greece, Netherlands, Spain, Sweden, UK).

Few studies engage with the question of which level of government should do what, bar a general focus on the need for decentralised and collaborative governance. One exception is Harries and Penning-Rowsell (2011: 188) on flood protection policy in England and Wales. They argue that the UK government should not delegate so much to its Environment Agency which follows the ‘fashion for public consultation’ then favours local victims and engineering projects which are not fit for long-term purposes, at the expense of ‘the needs of the wider population or to policy pronouncements by government’.

### What changes to policy or policymaking do climate researchers actually find?

The contrast between social justice and neoliberal approaches allows researchers to compare (1) a potentially positive focus on equity and justice, with (2) a tendency to interpret and implement policy through a neoliberal and technocratic lens that undermines structural approaches to equity and justice.

Sokołowski and Heffron (2022: 1–5) seek to explain failure through the lens of justice. Categories include:

- ‘inconsistency’, such as when a new government rejects policies generated in a just manner
- ‘hesitation’ or ‘lack of political will’, such as making commitments but not fulfilling them in time
- ‘wrong structure’, including a lack of assigned resources to deliver policy goals, or a tendency for policies to exacerbate problems such as fuel poverty
- a ’lack of coordination, soft regulatory approach, no enforcement, deregulation’, thus undermining the ability to hold – say - polluters to account, or change their behaviour
- ‘exclusion’, which undermines the mantra ‘no one is left behind’
- ‘nationalism’, or ‘ignoring the impact of policy in one country on other countries’.

The lack of goal fulfilment ‘can also be perpetrated by describing them in an ambiguous, constrained, or contradictory manner, with no measurable criteria, or timeline’ (2022: 4; see also Scobie, 2017 on fossil fuel subsidies in Trinidad; Leck and Simon, 2018 on the South Africa municipal government preference for election-friendly projects).

Many identify promising policy changes in rhetoric but limited progress towards social justice in practice. For example, high-level commitments to climate change meet with business-as-usual policymaking, or the language of inclusion provides a veneer for to help protect rather than challenge neoliberal approaches ( Copland, 2020; Crosweller & Tschakert, 2021). Huitema *et al*. (2016: 10) note that articles tend to ‘paint a disturbing picture of how climate change can compound existing inequities in both developed and developing countries’ (e.g. Hughes, 2013 and Benzie, 2014 on the lack of inclusion of ‘vulnerable’ or ‘disadvantaged’ groups in top-down adaptation processes).

***Limited UNFCC-led progress towards social justice***

Okereke (2006: 725) describes the UNFCCC and early COPs as an impetus for modest policy change, exhibiting ‘important normative shifts in the rule-structure of global environmental management’, which ‘have not proved momentous enough to generate policies outside of what the prevailing neoliberal socio-economic regime might permit.’. While early UNFCCC documents talk about equity:

‘the struggle remains largely contained by dominant forces which have co-opted the concept of justice for neoliberal ends. The result is that the core rules … continue to be underpinned by neoliberal conceptions of justice which privilege the interests of the political North. This suggests that the notion of global environmental justice has had a very limited success as a strategic resource for the developing countries in the counter hegemonic project of securing global intragenerational equity’ ( Okereke, 2006: 735).

In COPs up to and including Kyoto, Okereke (2010: 466) identifies a nascent focus on procedural justice but without defining just procedures. There are increasing references to ‘systemic injustice’ - such as relating to ‘historical patterns of inequity between the political North and South’ – but in the context of ‘the overriding commitment to the neoliberal philosophy with its emphasis on market liberalization’, which ‘limits the policy space and makes the pursuit of justice conceived in radical terms practically impossible’ (2010: 468). It remains unresolved ‘how justice concepts and principles might be translated into effective and politically feasible policies’, especially when the aim is for the Global North to help the Global South only if it is not too expensive and there is minimal discussion of historic responsibility and compensation (2010: 471; see also Klinsky, 2015; Lebel *et al*., 2018; London *et al*., 2013).

Many other texts present variations on this theme, such as to identify:

*Governments co-opting social justice to retain neoliberalism*. Alverio *et al*. (2021: 511–15) identify what ‘just planned relocation’ policies for climate crisis migration would look like, including ‘Recognition of affected stakeholders’, acknowledging the ‘causes of systemic injustice’ to prompt a fair redistribution of costs and benefits, and using ‘multiple measures of human well-being’ in cost-benefit analysis. They identify case studies (in India, China, Laos, Sri Lanka, Philippines, Lesotho, Mozambique, Australia, Solomon Islands, Fiji, Papua New Guinea, US, Panama) where policies or processes did not live up to those ideals (2021: 513). International organisations could seek to foster country level change, but have limited resources and rely on cooperation (2021: 519).

Multiple studies identify the co-option of the language of procedural justice to take forward neoliberal approaches and try to return discussion to the technical design of policy instruments, while giving minimal funding to aid participatory processes ( Baker, 2016 and Fløttum & Gjerstad, 2013 on South Africa; Bertana, 2020 on community relocation in Fiji; Johnson, 2021: 11 on Ghana; Copland, 2020 and Crosweller & Tschakert, 2021 on Australia and the US; see also Benites-Lazaro & Mello-Théry, 2019 on clean development policies in Brazil, Honduras, Mexico, and Peru;).

*The negative role of donors*. Wong (2020: 11) describes in Nepal a deliberate lack of attention to ‘Socio-political aspects, such as ensuring equity of access, social, and cultural acceptance’ to please external donors. Lebel *et al*. (2018: 442) describe in Lao PDR and Cambodia the knowledge among donor recipients that they need to play the game to increase their chances of funding while accepting that initiatives like ‘local participation in dialog events’ make little difference.

*Governments shuffling off responsibility for extreme events*. Crosweller and Tschakert (2021: 1) examine inquiries into major disasters - 2005 Hurricane Katrina (USA), 2009 Victorian Bushfires (Australia), and 2011 Queensland Floods (Australia) - to identify how actors narrated what went wrong, what worked, and what should be fixed. They identify the absence of the governance structures to help governments make decisions effectively while being informed by citizens (2021: 2–3). A common theme is the drawbacks of ‘neoliberal’ approaches to governance, where the role of citizens and stakeholders is (1) not valued during policy development and (2) used as a way to shuffle off state responsibility.

*Cautionary tales of false dawns*. London *et al*. (2013) track the California’s Assembly attempt to integrate ‘environmental justice’ and neoliberal climate change policies. While the ‘landmark’ Global Warming Solutions Act of 2006 set ambitious GHG reduction targets, the process involved ‘heated conflict’ between policymaker and environmental justice groups, largely because there was (1) a neoliberal focus on ‘cap and trade’ and a ‘rollback of strong state regulation of environmental quality and health’ (based on ‘market optimism’ and ‘state pessimism’), which (2) exacerbated injustices in relation to minoritization and racialised outcomes. Colenbrander (2019, on coastal protection) contrasts (1) a focus on procedural justice to produce just outcomes and foster citizen ownership (in South Africa where this principle is part of the constitution), with (2) the lack of co-governance, and default to top-down government.

*A lack of capacity or willingness to participate or deliberate at municipal levels*. Romsdahl (2020: 146 on the US and Australia) identifies the need for greater local capacity to engage in climate related deliberation,: ‘For many cities, their local governments face a variety of barriers to addressing climate change, including lack of resources and competing priorities, but perhaps most significant is the lack of capacity and willingness to even discuss the topic’. Brink and Wamsler (2018: 82) find that ‘Municipal administrations in Sweden rarely plan to explicitly involve citizens in local climate change adaptation’.

*Is climate injustice inevitable in authoritarian states?* Huang and Liu (2021: 1) compare approaches to energy ‘just transitions’ in China with other authoritarian and non-authoritarian systems. They expect authoritarian systems to produce unjust transitions because of the lack of procedural justice when the central government oversees command and control approaches and local governments govern via mandate. In each case study, ‘lower income social groups are more likely to be affected by the unintended consequences of transition policies’ and ‘the costs of energy transitions are disproportionately born by more vulnerable social groups’, while ‘procedural justice is hard to attain under authoritarian political systems … social injustice seems to be an inevitable by-product of energy transitions in authoritarian regimes’ (2021: 13).

***More routine implementation gaps***

In some cases, the explanations for limited progress relate to more general and expected limitations of policymaking, or lack of reciprocity between governments ( Buchholz *et al*., 2018: 191). A range of examples includes:

*Implementation gaps relating to opposition to policy change*. Sovacool *et al*. (2022: 1) compare the power and strategy of opponents to energy transitions in ‘carbon-intensive regions’ (Estonia, Germany, Greece, Poland, India, Norway, US) to help understand why governments do not live up to their modest plans to reduce GHGs (2022: 24). They identify the importance of context-specific political dynamics to explain what is going on, such as when ‘goals of energy transition are refracted through national and subnational institutions and through local mobilizations both in support of and opposed to those transitions’. Political processes also act as legitimate fora for politics and contestation: ‘community mobilization, protest, and social opposition matter not only because they can reflect democratic ideals and hold important decision-makers more accountable for their decisions, but also because they can impact energy security or result in lost revenues and even violence’ (2022: 1–2).

*Multi-level governance issues*. Gerlak and Schmeier (2014: 359) examine the use of river basin organisations to address ‘complex collective action problems’ by facilitating ‘dialogue around action to change’ (focusing on the Mekong River Commission, MRC, in SE Asia). They analyse MRC discourse from 2001–13, finding an emerging justice frame (e.g. on poverty alleviation - 2014: 369–70), but note the limits to its focus on ‘multilevel governance’, given the ‘historic disconnect between the regional and national decision-making landscapes in transboundary water governance (2014: 376–7).

*Limited clarity on solutions*. Linnér *et al*. (2012: 175) perform a prospective evaluation of some ‘incentives to promote sustainable development’ (‘Sustainable Development – Policies and Measures (SD-PAMs)’), finding that they ‘require a too intricate institutional framework to make it effective’. Designers were not clear on how the schemes should work (2012: 184).

*Inconsistent policy mixes*. Kaufmann *et al*. (2018: 325) identify a lack of debate on fair approaches to flood risk management (Netherlands): ‘flood risk or its management is only marginally discussed in terms of justice’. The combination of multiple schemes, administered by multiple bodies, with different rationales, produces well-funded policy but with unequal effects ‘depending on the type of flooding they are prone to, area they live in … or category of user they belong to (e.g. household, industry, farmer) (2018: 335).

*Incoherent policy mixes*. Hetz and Burns (2014) examine the role of urban planning in urban flooding (Johannesburg, South Africa). The right to housing is included in the constitution, a right not to be evicted is enshrined in legislation, and there are state subsidies for 3 million houses. However, there is a tendency to import ill-suited ideas from the Global North, which do not address informal dwelling and internal migration, as well to as ‘lock in’ policy ideas ill-adapted to modern problems of urban design. The ‘give away’ housing policy - so important to a post-apartheid South Africa, to address inequalities in the past - prevents policy changes that might alleviate the problem of housing on flood plains (2014: 890–2).

*Unexplained implementation issues*. Naeku (2020: 170) describes uncertainty about how to explain an implementation gap in Kenya. It has ‘progressive’ credentials as a supporter of procedural justice and as ‘one of the first in the African continent to enact robust climate law and policies that guide national and local action’, including multiple pieces of national legislation since 1999 and a Constitution which entrenches a ‘right to a clean and healthy environment as part of the fundamental human rights to which every Kenyan is entitled to’ (2020: 174–7). Yet, ‘disasters and extreme events have plagued the most vulnerable communities, particularly with the most affected being Indigenous People and marginalised local communities’.

*Gender implementation gaps in the delivery chain*. Acosta *et al*. (2019) explore the impact of the international norm of gender mainstreaming: ‘Uganda has not only succeeded in introducing a gender discourse in policies and development programs, but has actually become a point of reference for their gender mainstreaming efforts in the East Africa region’. However, there remains a ‘gender implementation gap’, where the ‘strategy has not yet yielded the desired results, as exhibited by the rampant levels of gender inequality in agriculture still prevalent across Sub-Saharan Africa and elsewhere’ (2019: 10). They explore if the language of GM is ‘lip service’, and if GM norms are less impactful than other norms found in local policy and practice (2019: 10). They find the GM language at national levels, but: ‘At the local level, where climate-specific interventions are to be implemented, the gendered discourses largely disappeared’ (2019: 12–13). This problem emerges in the context of ‘entrenched patriarchal cultural norms’, such as ‘the constraints that Ugandan women face in relation to access, control and ownership of productive resources, such as land. These constraints were framed by national and sub-national policy as being the result of engrained cultural beliefs and traditions’ (2012: 13).

*Limited learning from the unintended consequences of policy*. Koch and Verholt (2020: 507) identify the unintended consequences of REDD+ policy, particularly ‘the forest-focused payment for environmental services programmes’. Their review found examples of (1) ‘motivation crowding’, when people once motivated by conservation ideas now seek economic incentives, (2) ‘marginalization’, when policies reward individual land owners, and exclude ‘rural poor and sometimes specifically of women’, while ‘indigenous populations often do not recognize individual claims to land but take care of it collectively’, (3) ‘leakage’, when logging is banned in one area then increases in another, or prompts mining, (4) and ‘out-migration’ (2020: 514–15). They find some evidence of learning by organisations, but also *technical limitations* when organisations struggle to monitor and evaluate impacts, and *ideological limits to learning* in the UNFCC, Green Climate Fund, and World Bank. In other words, learning takes place through the lens of ‘a strong belief that market- and monetary-driven solutions are the best solutions’ (2020: 522–3).

***Learning from partial or uncertain successes***

Some researchers seek to understand the relationship between signs of initial success but uncertain futures, prompting scepticism about further implementation. Skjærseth (2021: 26) examines the puzzle of the EU’s progress despite a historic privileging of economic frames and the scale of opposition among member states. The 2019 EU Green Deal (EGD) comes with a commitment for net zero GHGs by 2050 as well as to “decouple economic growth from resource use and ensure social justice by leaving ‘no person or place behind’” (2021: 26). This deal received unanimous support, even among ‘the ten Central and Eastern European countries that joined the EU between 2004 and 2007’ and were ‘more concerned about energy security based on fossil fuels than climate change’ (2021: 31). However, many relevant environmental policy instruments have been slow to progress, and have not been well integrated with other sectors. One explanation for the disconnect is that EU actors were skilful at finding ways to promote politically feasible strategies with the flexibility for delivery in each state: ‘EU institutions have been instrumental in crafting policy packages that exempt and compensate the least climate-ambitious actors’ (2021: 38).

Zannakis (2015: 221) seeks to explain why Sweden went ahead of its EU GHG reduction commitments before 2009, in sectors such as ‘real estate heating’, ‘garbage dumps’ and agriculture. Zannakis (2015) identifies a temporarily successful confluence between two storylines - *Ecological Justice* (akin to civic environmentalism) which was supported by ‘Social Democrats, the Left party, the Green Party and some NGOs) and *Opportunity* (in ecological modernization) - which competed well with *Sacrifice* (this will hurt us economically, and other countries won’t follow). However, the latter storyline took hold when the centre-right took office in 2009 (see also Knaggård, 2014).

### How do they use policy theories to understand these dynamics?

Few texts use mainstream policy theories to explain policy and policymaking (beyond superficial citation). However, many draw on foundational research – largely by Hajer or Dryzek – that would generally be treated as pushing against the mainstream. Nevertheless, for our purposes, mainstream and non-mainstream approaches share two important insights that should inform the study of climate justice policymaking.

First, *treat policy analysis and policy process research as two distinctive but mutually informative fields of inquiry*. The former is research to inform the design of policy: what is the policy problem, and which solutions should governments choose? The latter is research on policymaking processes: why do governments define the problem that way, and why do they choose those solutions? ( Cairney & Weible, 2017).

Second, *treat policymaking as a necessarily political process involving many actors, not a technical project involving few policymakers and experts*. Mainstream and interpretive approaches treat old post-war (largely US and UK) stories of ‘rationalist’ policymaking as misleading and unhelpful. This story idealised a highly centralised and exclusive process of policy analysis, in which elite analysts translated science to produce one correct diagnosis of a problem and an optimal solution ( Enserink *et al*., 2013: 17–34; Radin, 2019). In its place, we find real world accounts that emphasise at least one of two elements:

*Better description*. Mainstream policy theory-informed studies identify a more complex policymaking environment, with many policy actors spread across multiple policymaking venues, competing to interpret and define a policy problem and propose solutions.
*Better prescription*. Critical approaches reject the argument that the old rationalist story was an ideal to aspire to. Rather, the processes to inform problem definition and the generation of feasible solutions are – and *should be* – political because they necessarily involve the translation of knowledge, beliefs, and values into policy. Therefore, greater citizen and stakeholder participation should challenge parochial, elitist, and exclusive conceptions of policy processes, and greater deliberation should challenge the tendency to (wittingly or unwittingly) reproduce the same damaging ideas ( Cairney, 2021a).

In that context, Hajer (1995: 2) uses discourse analysis to identify the ‘social construction of environmental problems’, which includes not only ‘what is being said’ but also ‘the institutional context’ which helps to determine the meaning of speech and action. This context helps to explain why, in an argumentative contest, some understandings of problems dominate and are ‘seen as authoritative, while other understandings are discredited’ (1996: 44; Hajer & Versteeg, 2005). The management of environmental policies takes place in a wider context in which the state protects and reinforces capitalist ideas and rules, treating current institutions as a source of solutions to (rather than inevitable causes of) environmental problems, thus placing profound limits on the feasibility of new approaches and solutions ( Hajer, 1995: 3).

In particular, the idea of ‘sustainable development’ fostered by the Brundtland report in 1987 could be seen as (1) a challenge to the status quo or (2) a ‘rhetorical ploy’ to fit environmental policies into a neoliberal paradigm that favours economic development over sustainability (1995: 12). Similarly, ‘ecological modernization’ sums up the general idea that ‘existing political, economic, and social institutions’ can be reformed to protect the environment by: using science to determine the size of the problem and the costs and benefits of action; fostering collective action to produce sustainable policies that benefit society; and, treating economic growth and environmental sustainability as reconcilable rather than contradictory (1995: 25). This idea flourishes partly because it is regarded as politically feasible in neoliberal contexts. For example, it seeks to replace state and business conflict over the costs of pollution with cooperation to identify win-win scenarios and develop long-term strategies. At the same time, its status remains contested: as one step in a reflexive ‘modernisation’ journey towards environmentalism, or a mere façade to prevent progress (1995: 26–34).

Similarly, Dryzek (2022: 9–10) describes discourse as: ‘a shared way of apprehending the world’, with storylines resting on ‘assumptions, judgments, and contentions’ that are essential to communication because they ‘provide the basic terms for analysis, debates, agreements, and disagreements’. Further, each discourse is ‘bound up with political practices and power’ as well as ‘some material political realities’, such as if states are obliged to foster capitalism and economic growth if they seek investment from multi-national corporations (2022: 9–10). Dryzek (2022: 14–7) compares four competing ‘environmental discourses’ according to the extent to which they (1) seek to depart from ‘industrialism’ in a pragmatic/ *reformist* or *radical* way, and (2) treat current industrial reality as a given ( *prosaic*) or seek to imagine a different reality ( *imaginative*):

*Limits, boundaries, and survival* is radical and prosaic, seeking radical adjustments to current practices on the assumption that the Earth has a finite capacity which should place limits on (say) the size of the human population (2022: 27–72).
*Problem solving* is reformist and prosaic, seeking pragmatic ways to modify current practices, while putting faith in (a) experts to diagnose and solve the problem, (b) markets to provide incentives for environmental action, and (c) citizens to keep them on their toes (2022: 73–146).
*Sustainability* is reformist and imaginative, taking pragmatic steps towards a new reality, such as by putting faith in experts and markets to find new sustainable ways to maintain development (akin to sustainable development and ecological modernisation) (2022: 147–185).
*Green radicalism* is radical and imaginative, seeking radical change towards a new reality, in a variety of ways, including ‘ecological justice’ and ‘ecofeminism’ (2022: 187–222).

In that context, researchers can identify the extent to which political action – such as by political parties, social movements, or elected policymakers – is taken in the name of storylines associated with each environmental discourse (2022: 223–32) or the ‘gray radicalism’ backlash against environmentalism (2022: 233–47).

Multiple studies use such insights try to pinpoint the discourses, processes, actors, and coalitions that are getting in the way of progress towards climate justice. They include studies of framing (largely in media discourse), socio-technical systems, political theory (to inform discussions of justice), and discourse analyses (to identify a battle of ideas or competing political movements).

***Addressing uncertainty and ambiguity***

Drawing largely on non-mainstream policy theories, many texts provide an equivalent to the mainstream study of ‘bounded rationality’ (see **Discussion**), to document the exercise of power to retain a dominant and damaging understanding of climate issues ( Huitema *et al*., 2016: 4). In particular, they criticise two political acts: (1) to rely too much on scientific or academic knowledge, contributing to procedural and recognitional injustice; (2) to support a damaging neoliberal frame, and pay insufficient attention to social justice, when describing and addressing climate change, contributing to distributional injustice. Examples include:

*The battle of ideas (mitigation)*. McGee and Taplin (2009: 214) describe (in the early 2000s) a ‘contest of ideas for the future of the international climate change regime’, between (1) UNFCCC-led policies and (2) those of countries like the US and Australia favouring more voluntary targets for countries and businesses, with both largely ignoring social justice frames.

*The battle of ideas (adaptation)*. Lebel *et al*. (2018: 429–30) describe two competing frames regarding climate change adaptation (Cambodia and Lao PDR):

a ‘techno-managerial frame’ talks up the value of ‘technical, infrastructure or management solutions’ and ‘often serve more to protect than to challenge the current development paradigm’
‘the sociopolitical frame’ seeks to reflect ‘on social injustices and inequities, as well as suggesting solutions that are more likely to politically reorder society’, such as ‘social protection schemes, (re)distributive land and resource policies, as well as support for processes that could potentially equalize structures of representation and power’.

Frame use varies by country, with authoritarian countries more likely to use technical language and democratic countries ‘may encourage more diverse or inclusive framings of adaptation that gain legitimacy through deliberation or accountability relations’ (2018: 30). However, their main focus is on the role of international donors in perpetuating the techno-frame while co-opting the socio-political frame and disguising unequal power relations. Organisations seeking funding become aware that: ‘Saying the right things about integration, mainstreaming, gender, and engagement … improves acceptance of projects’, and that they can emphasise ‘local participation in dialog events or other activities, without relinquishing control of key decisions’ (2018: 442).

*Limited attention to equity*. Gebara *et al*. (2017: 213) analyse national media frames to identify ‘the nature of the contested domains of the REDD+ policy process’ in Brazil, finding that a focus on equity – such as regarding ‘land conflicts and unfair benefit-sharing’ - is small. Liebenguth (2020: 189–90) explores the discourse of energy security in the ‘water-energy-food (WEF) security nexus - an integrative approach to sustainable development with significant global reach’. An ‘economic productivity’ discourse dominates, to focus on how to maintain productivity and growth when these resources are scarce, thus marginalising ‘environmental justice’ frames focused on ‘challenging the notion of green economic growth and bringing to the fore critical issues of access and allocation such as food sovereignty, the right to water, and energy for all’ (2020: 193).

*Unresolved ambiguity and imbalances of power*. Alexander *et al*. (2018) identify unresolved ambiguity regarding the meaning of legitimacy in the governance of flood risk. Bertana (2020: 903) describes a misplaced tendency to focus on community participation in policymaking without highlighting inequalities of power. The latter are inherent in processes to reduce uncertainty (organisations partially withholding information) and ambiguity (organisations limiting ways to understand the problem).

*Obstacles to audience receptivity to social justice frames*. Schmidt and Schäfer (2015: 535) identify contested notions of climate justice (in German, Indian and US media), including 5 ideal types in media coverage: (1) climate change is exaggerated, so state limits on personal freedom are unjust, (2) climate change is real, but can be solved via modifications to the market, (3) states have a moral duty to intervene more, (4) ‘Climate justice means that taking action is a moral obligation of those historically responsible for causing the problem’, (5) climate policies are positive if they provide value to businesses and individuals (2015: 543). In that context, studies examine what influences receptivity to social justice frames, including:

Betz (2015: 555) argues that competing discourses may be as much about debating who has scientific authority as norms/values.
Kleinen-von Königslöw *et al*. (2019: 518–21) focus on the need for national governments and other actors to legitimise the d agreements (and the UN-led governance/ architecture) and encourage or oblige their uptake among citizens and businesses. They find a tendency in each national context - Australia, Brazil, Germany, India and the United States – for coverage to be dominated by national figures who tend to be ‘not impressed with climate regimes’ (2019: 534).
Fairbrother *et al*. (2021: 1–3) examine ‘political trust’ in surveys/ experiments in Sweden, Spain, South Korea, and China, arguing that if people think that policymakers are ‘dishonest, corrupt, and/or incompetent’, they will ‘doubt that their government can or will design and/or implement policies in societally beneficial ways’.
Knox-Hayes *et al*. (2013: 610) find that people in countries more dependent on securing imported fuel are less concerned with its equitable distribution.
Songsore and Buzzelli (2016: 15) find that local newspapers amplified concerns on the negative public health effects (felt unequally) of onshore wind turbines near homes.
Wardekker *et al*. (2009) examine the US Christian discourse regarding support for climate change action, identifying three moral storylines that ‘may find support among the US population’: ‘conservational stewardship’ (conserving the ‘garden of God’ as it was created), ‘developmental stewardship’ (turning the wilderness into a garden that it should become) and ‘developmental preservation’ (God’s creation is good and changing; progress and preservation should be combined).
In contrast, Severson and Coleman (2015: 1277) find that ‘Religious moral frames and economic efficiency frames are ineffective, whereas scientific frames, secular moral frames, and economic equity frames are effective at increasing overall policy support’, while ‘the positive science frame and economic equity frame reduce the ideological divide in climate policy support’.
Lehotský *et al*. (2019: 774) identify a tendency for environmental frames to have limited influence on media debates on coal production and consumption in the Czech Republic, since many communities have strong connections to mining, and the fate of coal companies is described as an economic problem rather than climate opportunity (yet, ‘the Czech Republic has a unique, 28-year-old history of coal-restricting policies motivated by environmental reasons’ - 2019: 775).

***Dealing with ambiguity: how should researchers or activists respond?***

Should advocates of climate justice treat actors such as fossil fuel companies as the enemy, to help challenge a dominant discourse? Should they treat actors such as policymakers as potential allies and work within the current discourse? Mangat *et al*. (2018: 187) describe the former, using discourse analysis to identify the main narratives associated with fossil fuel divestment movements in North America (e.g. 350.org in the US). They identify ‘war’ as the dominant narrative, to signal that this movement sees fossil fuel companies as the enemy rather than a sector with which to find consensus:

‘By polarising climate action and identifying an antagonist against which to mobilise, divestment discourse has articulated climate change as an explicitly political phenomenon, in contrast to the primarily consensus and collaboration-based approaches that have predominated in climate politics’ (2018: 201).

Muncie (2021: 537) describes the latter, identifying the frames used by campaigners seeking fossil fuel divestment by the Scottish Parliament and government: ‘(1) Financial Risk and Economics’ (there are better investments elsewhere), ‘(2) Climate Justice, Morality and Ethics’ (fossil fuel extraction exacerbates the unequal costs and benefits of development) and “(3) ‘Climate Emergency’ and Urgent Action” (along the lines of the IPCC) (2021: 539–41). In contrast to 350.org, campaigners in Scotland largely avoided confrontation (2021: 545). This approach boosted cooperation but presented mixed signals of intent:

‘On the one hand, a frame of climate justice demonstrates divestment’s commitment to more radical aspirations and a holistic approach to tackling climate change, while on the other hand, a finance frame perpetuates notions of ecological modernisation and market solutions’ (2021: 545).

Daub’s (2010) case study of the Communications, Energy and Paperworkers Union (CEP) in Canada shows that it is not inevitable that they would support their industry in opposing stronger climate change measures (e.g. preventing deforestation). The union leadership (1) oversaw a collaborative internal process, to encourage union members to address GHG reduction as citizens rather than vested interests, while (2) using an ‘environmental justice master frame’ to counter industry frames, which also helped the CEP avoid being seen as a ‘dinosaur’ (2010: 134–5).

Sakellari (2022) calls for a similarly critical approach among journalists to challenge a tendency in Western media to portray climate migration in relation to ‘security threat’ (the host country becomes less safe), or ‘victim’ (to emphasise their vulnerability and promote a victim-saviour narrative). Rather, emphasise the role of migration as rational adaptation to the climate consequences that they did not cause and cannot control (2022: 74–9).

***Theory-informed accounts: multiple streams, advocacy coalitions, and new institutionalism***

*Multiple Streams Framework (MSF)*. Two accounts use multiple streams analysis, which suggests that major policy change may only occur during a ‘window of opportunity’ when three conditions are met: attention to the problem is high (problem stream), there already exists a technically and politically feasible solution (policy stream), and policymakers have the motivation and ability to select it ( Kingdon, 1984; see also Tanner *et al*., 2019).

Da Conceição *et al*. (2015: 243) examine why the policy instrument ‘payments for environmental services’ (PES) is adopted slowly despite its potential to encourage forest conservation. They use the MSF to analyse the adoption of PED in Brazil, Ecuador and Peru, finding common elements:

Problem stream. Policymakers paid low attention to deforestation, but were relatively committed to forest conservation schemes that addressed poverty in relevant areas.
Policy stream. Environmental agencies are not powerful enough to lead policy design, allowing non-environmental agencies and groups to produce incentive schemes with dubious environmental impacts.
Politics stream. In Brazil, governmental stability – plus high attention – kept windows for policy change open for longer, albeit without prompting evidence-informed conservation policy to emerge. In Peru and Ecuador, policy change was designed centrally and from the top-down, without much need to capture wider political feasibility (2015: 250–1).

Gray and Bernell (2020: 1) use the MSF to explain the rapid adoption in 2016 of ‘the Oregon Clean Electricity and Coal Transition Plan, which doubles the state's renewable portfolio standard to 50% and eliminates the use of coal-fired electricity by 2030 (2020: 1). In terms of the window of opportunity: there has been high attention in Oregon to climate change and the role of fossil fuels (problem stream); multiple initiatives are in place or have been proposed before (policy stream); and, there was pressure from environmental and other groups to pursue more ambitious targets (via the Ballot system), which had general public support, plus coal was a small and dwindling part of energy anyway (politics stream). The window opened when this alliance saw the chance to pass a bill during a 5-week legislative session (2020: 6).

*The Advocacy Coalition Framework (ACF)*. The ACF describes actors entering politics to turn their beliefs into policy, forming advocacy coalitions with like-minded actors ( Jenkins-Smith *et al*., 2018; see Discussion). No account uses it in a recognisable way (although Malloy and Ashcraft, 2020 try to adapt it, while Pellegrini-Masini *et al*., 2020: 5–6 suggest that equality could be a motivational ‘deep core belief’). Yet, multiple studies identify comparable issues that could inform more systematic applications.

For example, Basseches *et al*. (2021: 23) find that there can be a broad coalition in favour of climate change policy but containing different coalitions debating how to get there. In the case of California, they identify:

‘a coalition of “market-oriented” environmental social movement organizations (SMOs), who allied with private corporations to advance market-friendly climate policy’ (currently the more powerful coalitions)
‘a coalition of “justice-oriented” environmental SMOs, who viewed capitalist markets as the problem and sought climate policy that would mitigate the uneven distribution of environmental harms within the state’ (with the potential for greater future influence).

Their case raises the question: which environmental activists are allies or opponents when they engage with each other (and other actors) on concrete measures? They raise the dilemma of ‘strange bedfellows’, where environmental groups debate whether to cooperate with businesses to foster pragmatic policy change (with a clear request to government), or treat them as the enemy of radical change (which is harder to define).

*New institutionalism*. New institutionalism (NI) is a broad description of multiple approaches to the study of formal and informal rules in policymaking ( Cairney, 2020). It may include, for example, a government’s rules of engagement – to promote more or less inclusivity - with environmental groups ( McCauley, 2015: 274).

Pillai and Dubash (2021: S93) use NI to examine climate politics and policy in India, describing its tendency to operate ‘under the electoral radar’. A lack of policy progress relates to two main factors. First, ‘an entrenched narrative frame that resists emission reductions’, linked to path dependence in relation to ‘a coterie of bureaucratic elites, civil society, and legislators’ reproducing or reinforcing ‘the principle of Common but Differentiated Responsibilities (CBDR) and respected capabilities’ relating to India’s relatively low emissions per capita. Second, India’s ‘hierarchical inter-ministerial bureaucratic politics’ produces ‘a climate politics characterized by opportunism’, in which a focus on development is the norm but mitigation may have occasional traction. This approach fosters the ‘processing of climate policy across multiple ministries without radically altering their priorities … characterized by climate nodes spread across government, stitched together by relatively weak and unstable cross-ministerial coordination and strategy bodies’ (2021: S94). They describe path dependence but the opportunity for policy change linked to a tension between ‘seeking international legitimacy and the domestic constraints of poverty and sensitivity to energy prices’. The latter prompts policy and policymaking changes - to take mitigation and adaptation more seriously across government - but largely by ‘layering’ a new and poorly resourced strategy (National Action Plan on Climate Change, NAPCC) onto a system that is not conducive to its delivery (2021: S111).

*Power*. Sova *et al*. (2015: 463–4) is the only article to draw on theories of power. They identify the UNFCC’s influence over ‘highly vulnerable small-scale farmers’ in relation to Lukes’ (1974) three dimensions of power:

- Visible contests with winners and losers. Some actors – such as China – succeeded in ‘opposing agriculture’s place within a binding UNFCCC agreement’ (2022: 466).
- Agenda setting. Developing countries have had some influence since they are supported to contribute to UNFCC debates on climate adaptation, but the process is not well resourced, and many key decisions take place outside of the formal arena (2022: 467).
- Manipulation of beliefs or preferences. There are ‘three sources of preference shaping power’: (i) the dominant discourse privileges scientific elites, not wider participatory processes; (ii) scientific elites, including the IPCC, favour a narrow range of technological fixes, with smallholders not aware of the wider possibilities; (iii) formal debates describe ‘poor countries and not poor people’, producing intentional or unintentional exclusion of diverse voices (2015: 467–8; see also Sovacool and Linnér, 2016: Chapter 5).

## Discussion

Engagement with policy concepts and theories is important because policy processes are essential objects of study in their own right. Put simply, climate justice research may engage with political reality, but it also needs to engage with the realities of policymaking or policy processes. Empirical studies, guided by policy concepts and theories, help to identify the routine patterns or dynamics of policymaking that are present regardless of the specific politics of the day. They identify features of policymaking that are general rather than specific to climate policy, such as a tendency towards limited policy change, an in-built need for policymakers to ignore most issues and evidence, and a complex policymaking system (or policymaking environment) over which they have limited knowledge and even less control. In that context, we present the following three core insights based on our synthesis of policy theories and empirical studies on the nature of policy change, bounded rationality, and policymaking complexity. We argue that all three broad insights should guide further interdisciplinary research in relation to climate justice and policymaking, but only find meaningful engagement with one (regarding bounded rationality).

### Major policy change is rare and difficult to predict

Almost all relevant texts provide a general discussion of the kind of climate justice they would like to see. However, it is difficult to find discussions of how much policy change we would expect to find, based on how policy changes, and how policy processes constrain or facilitate change (with some exceptions, such as Rootes *et al*.’s 2012 brief account of NI to explore if gradual institutional changes can eventually be transformational). Examples of theories or concepts to inform future development include:

*Punctuated equilibrium theory* ( Baumgartner *et al*., 2018). The sum total of policy changes is a combination of a huge number of small changes and very small number of major changes (which are far easier to measure than predict). If so, what would cause the transformational changes sought in most articles? In PET studies, policy change is largely a function of ‘disproportionate information processing’, where policymaker attention to information about problems bears limited relation to a problem’s size or the availability of information. In classic studies, attention relates to the ability of some actors to monopolise how policy problems are framed and the means to respond, prompting others to shop around for venues that contain more sympathetic audiences (e.g. Baumgartner and Jones, 2009 provide case studies of major change after several decades of continuity). In modern studies, transformational change often relates to a bandwagon then pressure dam effect, in which the high levels of attention required to overcome ‘institutional friction’ may contribute to a short and profound burst of activity in which more actors are involved in debates on how to frame and solve issues.

*Advocacy coalition framework* ( Jenkins-Smith *et al*., 2018; Weible & Ingold, 2018). The ACF describes actors entering politics to turn their beliefs into policy. They cooperate within advocacy coalitions of like-minded actors and compete with other coalitions. This activity takes place in policy subsystems devoted to specific issues or sectors, in policymaking environments that constrain or facilitate each coalition. Subsystem activity can be low salience and technical, amenable to brokering agreements. Or, it can be high salience and competitive, characterised by conflict in which actors romanticise their own cause and demonise opponents. In that context, policy change may result from several sources, including: (1) routine policy-oriented learning through the lens of a coalition’s beliefs; or (2) non-routine change prompted by ‘shocks’, associated with an external influence such as new government or social, economic, or environmental crisis, or internal dynamics such as actors questioning their beliefs following policy failure.

*Policy paradigms*. Hall’s (1993) famous account of policy change identified three types: first order describes routine bureaucratic changes to instruments, second order describes non-routine changes (such as using different instruments) while maintaining policy goals, and third order relates most closely to the transformations required for climate justice. The latter may only happen following a crisis of policy failure that policymakers cannot solve or explain with current ideas. It prompts a reappraisal and rejection of the dominant ‘policy paradigm’ in favour of new ideas and the reliance on new sources of advice. More recently, multiple studies engage with Hall’s analysis to suggest that this dynamic did not happen in the way that Hall suggested, or is remarkably rare, with the more likely route towards transformation coming from a series of more gradual changes (see Cairney, 2020).

Overall, such insights help to identify the mechanics or processes of policy change and ask important questions about transformations. First, is a pressure dam effect likely in relation to climate justice, and what would the process look like? Second, should policy actors – driven by beliefs associated with climate justice – seek to cooperate with allies and demonise opponents? Third, is radical change only likely via a series of changes, and - if so - how would researchers know if current changes were part of that trajectory rather than a means to slow it down (see also Buch-Hansen & Carstensen, 2021)?

### Bounded rationality prompts policymakers to ignore most problems (and evidence) and reject most ways to interpret them

*Bounded rationality* describes the cognitive and organisational limits to gathering and processing policy relevant information ( Simon, 1957: 198). Policy studies distinguish between two political acts to respond to bounded rationality:

- Policymakers use evidence selectively to reduce *uncertainty* (a lack of knowledge regarding a policy problem or solution).
- Policymakers pay attention to - or propose or support - one frame or problem definition to reduce *ambiguity* (the potential to interpret problems in competing ways) ( Cairney *et al*., 2016: 399; Majone, 1989: 8, 21; Zahariadis, 2007: 66).

Policy studies relate ambiguity to the exercise of power to get what actors want, such as by framing issues to draw attention to one understanding at the expense of the rest (e.g. Baumgartner & Jones, 2009; Knaggård, 2014). In that context, while few climate justice studies use the phrase ‘bounded rationality’, there are parallels with the focus on dominant discourses in climate justice research, which argue that policymakers:

- Rely too much on scientific or academic knowledge when seeing to reduce uncertainty (contributing to procedural and recognitional injustice)
- Support a damaging neoliberal frame, and pay insufficient attention to social justice, when seeking to reduce ambiguity (contributing to distributional injustice).

In some inequalities policy research, ambiguity is examined in two ways: (1) as a problem, since it is difficult to translate vague aspirations into concrete action; or (2) as an opportunity, to use ambiguity creatively to generate initial support or to foster continuous contestation to keep an issue high on the agenda ( Cairney *et al*., 2022a). One included article focuses on these potential benefits. Werners *et al*. (2021: 168) review the literature on sustainable development to see which ‘climate resilient development pathways’ are described as feasible. They identify a plethora of terms – including ‘climate compatible development’, ‘triple wins for mitigation, adaptation and development’, and ‘low carbon resilient development’ – and uncertainty regarding how to translate abstract concepts into concrete action (2021: 169). For example, do people describe ‘resilience’ positively, does ‘pathway’ describe a metaphor or specific sequence of steps, and what would transformed systems look like? (2021: 170–1). This ambiguity can be a good thing if it fosters ‘the potential to create deliberative spaces … climate resilient development pathways can accommodate multiple, diverse visions and consider the deep uncertainty that exists when exploring possible futures' (2021: 173).

### Policymakers operate in a complex policymaking environment of which they have limited knowledge and control

Policy theories seek to conceptualise policymaking in relation to two main perspectives ( Cairney, 2020). First, *complex policymaking systems* exhibit: positive and negative feedback (the same policy action can have a minimal or maximal effect); sensitivity to initial conditions (events and choices in the distant past can create path dependence), and strange attractors (patterns of behaviour can endure for long periods despite the ever-present potential for disruption). This perspective helps to explore a tendency for policy practices or outcomes to ‘emerge’ in the absence of single central government control, and perhaps encourage more feasible and adaptive behaviours focused on local activity in multiple policymaking ‘centres’. Second, in different ways, most theories narrate the importance of *complex policymaking environments* consisting of: many policymakers and influencers spread across multiple venues (or centres of authoritative choice), with each venue enjoying its own informal and formal rules, networks, dominant beliefs, and responses to socio-economic conditions.

Crucially, these conceptual discussions relate largely to the study of *individual countries* that exhibit emergence or polycentricity regardless of the presence of an authoritative central government. The task for scholars of environmental policy is to consider how the international arena fits in, such as to rule out the – already limited – ability to rely on a small number of authoritative ‘centres’ to drive much-needed policy change.

In that context, two included texts identify the concepts that should be used to greater effect. First, Huitema *et al*. (2016: 7–8) focus on the importance of ‘polycentric governance’ and the Institutional Analysis and Development Framework (IAD) (citing Ostrom, 2010). It explores the absence of a single authoritative ‘centre’ of power to direct policy, and the presence of many autonomous or semi-autonomous organisations who need to collaborate to produce and deliver common aims. On the one hand, this system is an obstacle to straightforward routes to necessarily radical policy changes. On the other, it opens up the possibility for (1) innovation and learning, and (2) the co-production of policy by many actors in many sectors (not subject to external authority) (2016: 7–8).

Second, Burch *et al*. (2019: 6) outline the ‘earth system governance’ framework to explore the ‘governance arrangements that prompt or support the – societal, economic, and technological - transformations necessary to address climate change, as well as the required or actual transformations in climate change governance, such as away from top-down international approaches towards more autonomy and accountability in relation to states and substate actions’. It also highlights the ‘diversity in norms and knowledge systems’ that preclude a single agreed solution to the problems of earth system governance (2019: 6). Policymaking is fragmented and complex, containing many contested values and ideas regarding climate change and justice, how to organize political action, who should win or lose from policy, and how multiple centres should act collectively to respond to crisis (2019: 8–13).

Such concepts help to highlight an uneasy relationship between what researchers and policy actors may identify as:

- *Necessary*, such as governance mechanisms to encourage a transformation towards sustainable environments, and a just transition towards sustainability, or
- *Likely*, which is much less certain given the distribution of responsibilities and wide range of beliefs and practices across the globe.

Such issues are touched upon by multiple articles, but with fleeting reference to the IAD ( Anderson & Schirmer, 2015; Buchholz *et al*., 2018; Crosweller & Tschakert, 2021; Häyhä *et al*., 2016; Jerneck, 2018; Macintosh, 2013; Mani *et al*., 2021; Okereke, 2006; Rosen, 2015; Sarkki *et al*., 2017; Schmidt & Schäfer, 2015; Sovacool *et al*., 2020; Valdivia & Balcell, 2022). There is minimal attention to the evidence that polycentric governance or more decentralised and participatory processes help to deliver environmental sustainability or climate justice policies ( Morrison *et al*., 2019; Newig & Fritsch, 2009).

There is also minimal attention to the *politics* of policy coordination or collaboration, in which included texts seek vague aims such as ‘integration’ without acknowledging that policymaking ‘silos’ make sense and are often fiercely guarded, even within the same central government. We make this point in our public health review:

Most policy is processed in silos that seem to defy central coordination. Silos develop rules appropriate to their own contexts, and their logics do not change simply because the overall effect looks like uncoordinated and incoherent policymaking … policy studies present them as ever-present forces to which to adapt. A lack of intersectoral action seems incoherent to some but *makes sense* to others' ( Cairney *et al*., 2021: 24–5).

As Cejudo and Trein (2023: 9; 12) argue, ‘policy integration is in permanent political tension with the sectoral logic of policymaking’. The pursuit of integration is not a technical problem or ‘a single moment when those tensions are solved once and for all’, but a continuous ‘political process’ to address the fact that policy actors operate according to the rules, norms, or logic associated with their ‘own policy subsystems’ logic, ‘and undermine the efforts toward policy integration at every moment of the policy process’ (2022: 4; see also Cairney, 2021b; von Lüpke, 2022).

More generally, few texts engage with the politics of policymaking, such as when policymakers in democracies have to juggle different measures of policy success. In that context, one included text highlights the limitations to a focus on policy failure (1) from the perspective of researchers, without (2) recognising the value of interpreting success and failure through the eyes of the policymakers they study. Newman and Head (2015: 342) call for greater precision when describing failure, including categories such as: it did not meet stated objectives; it caused inappropriate outcomes (such as to exacerbate inequalities); it damaged an actor politically; or, it was not implemented well (2015: 345). The value of the expansion of categories is to note that: (1) policies can have multiple stated/unstated objectives; (2) policymakers can have multiple – policy and political - objectives; and (3) policy evaluations may reach different conclusions at different ‘stages’ or over time (2015: 345–50).

Their case study is ‘Australian climate change policy’ under Rudd and Gillard governments (2007–13), often treated as both a policy and political failure by environmental activists because it did not go far enough and still undermined the authority of the government. Rather, Newman and Head (2015) argue that the government met policy objectives related to the Kyoto agreement, while the lack of effective climate action did not hurt Rudd and Gillard because it is not a widely supported move (especially during a financial crisis). This analysis ties to a wider literature on the political evaluation of policy success, which reminds us that the types of success most discussed by climate justice researchers are – programmatic (policy is appropriate and works as intended) and process (policy is legitimised via procedurally just processes). This focus is incomplete without a proper consideration of political success (the policy does not undermine support for policymakers) ( McConnell, 2010). There is a misplaced tendency to treat politicians as corrupt and electoral politics as an inconvenience rather than an essential part of the democratic process underpinning the pursuit of justice.

## Limitations

No search or review is comprehensive, and it is possible that a larger series of searches for specific organisations (such as UNFCCC) or sectors (such as energy or agriculture) could have yielded further results. Rather, we used a general keyword search, combined with manual inclusion processes, to immerse ourselves in the wider subfield of climate justice and avoid biased searches through the lens of previous reviews in health and education. We also used snowballing to make sure that we were aware of common reference points in this field. Nevertheless, as Brandstedt’s (2023) peer review notes, our search rules excluded the direct examination of ‘climate ethics’ research that engages with ‘normative ideals of climate justice’ and ‘the political reality around climate policy-making’ (although many of the included articles engage with those concerns, while some - such as Sovacool *et al*., 2017 – cite Caney and colleagues directly). As a result, we have engaged relatively sparingly with key concerns in the wider research field, such as how to conceptualise climate justice and injustice (an important exercise in its own right). 

As with each of our reviews, the starker limitation relates to the bias in research towards Global North researchers and experiences. We did not restrict our search geographically, but our requirement for publication in English strongly influences the results. There is a greater spread of countries in this review, compared to our previous reviews in health and education, but the list of included texts is not representative of global experience. Therefore, the Results and Discussions sections have clear implications for policy and policymaking, but their application is not universal.

## Conclusions

Environmental scholars have a clear sense of the general problem that they face: climate change represents an existential crisis that requires rapid and radical policy change, but too few policymakers pay high and sustained attention or deliver a proportionate policy response. Scholars of climate justice also identify the unequal and unfair consequences: some people and countries are disproportionately responsible for the actions that exacerbate climate change, while other people and countries shoulder an unequal burden as a result.

Climate justice scholars present a common narrative, as follows. First, there is disproportionately low attention to climate justice even when attention to climate change is high. For example, the IPCC and UNFCCC exhibit far more clarity and ambition in relation to climate change and make relatively vague and non-committal moves towards climate justice. Second, this lack of energy for reform relates to a losing battle of ideas. Most researchers seek social justice built on recognitional, procedural, and distributive forms of climate justice, but find that a neoliberal alternative dominates most policy agendas. The effect is to reinforce a focus on market and voluntary solutions, with few governments willing to redistribute power or economic resources. Third, there need to be radical changes to policy and policymaking to transform the economy and society while addressing climate change. Fourth, there is a major gap between what is required and what happens. Fifth, these problems prompt debates among climate justice scholars (and activists) about how to respond. One classic dilemma is how to portray actors who do not share their beliefs: as enemies of progress towards rapid transformation, or potential allies to securing more feasible, gradual, and likely changes.

Overall, we find a large number of texts contributing to the rich description of problems with policy and policymaking, with particular strengths in relation to the study of dominant discourses and the absence of procedurally just processes. However, most texts engage superficially with mainstream theory-informed studies of politics and policymaking. The effect is that almost all scholars know what they need from policy processes, but do not describe how those processes actually work or the extent to which they are likely to produce the required effects. Most environmental scholars still treat the policy process as a ‘black box’ that is largely beyond our understanding ( Biesbroek *et al*., 2015; Morrison *et al*., 2019).

While theory-informed studies do not solve the problems raised by climate justice scholars, they provide the concepts or language to help identify patterns and mechanics of policy change, which should help to raise research questions pertinent to the ongoing study of climate change action. First, since major policy change is rare, what would it take to happen and would we know it if we saw it? Second, what would it take to prompt lurches of attention to new ideas and sources of information, and is high ambiguity a problem to be solved or opportunity to be exploited? Third, what can we learn from studies of complex policymaking systems or environments? In particular, do frameworks such as the IAD help us to understand how policy actors collaborate to produce procedurally just processes with benefits for natural and other resources?

## Ethics and consent

Ethical approval and consent were not required.

## Data Availability

All data underlying the results are available as part of the article and no additional source data are required. Open Science Framework: Climate change and equity: a qualitative systematic review of the role of policymaking in just transitions. https://doi.org/10.17605/OSF.IO/SZP9X ( Cairney & Timonina, 2022) This project contains the following extended data: Structured bibliography Qualitative Systematic Review – Climate Change Policy 6.1.23.docx (List of 108 included texts, according to each database). OSF Protocol Cairney Timonina Qualitative systematic review climate justice 3.11.22.docx (Study protocol). Open Science Framework: PRISMA checklist for ‘How can policy and policymaking foster climate justice? A qualitative systematic review’. https://doi.org/10.17605/OSF.IO/SZP9X ( Cairney & Timonina, 2022) Data are available under the terms of the Creative Commons Attribution 4.0 International license (CC-BY 4.0).
