# Supplementary material for: How can policy and policymaking foster climate justice? A qualitative systematic review
Source: Open Res Eur. 2023 Oct 10;3:51. Originally published 2023 Mar 31. [Version 2] doi: 10.12688/openreseurope.15719.2 (PMC10724653; doi:10.12688/openreseurope.15719.2)
Supplement: Peer review history

# Reviewer report: Reviewer response for version 2

**DOI:** 10.21956/openreseurope.17995.r35547

**Published:** 2023-12-15

**Authors:** Saba Hinrichs-Krapels

**Affiliations:** 1 Faculty of Technology, Policy and Management, Technische Universiteit Delft, Delft, South Holland, The Netherlands

Thank you for this revised version of the manuscript. The authors have addressed all of our points thoughtfully.

Are sufficient details of the methods and analysis provided to allow replication by others?

Yes

Are the rationale for, and objectives of, the Systematic Review clearly stated?

Yes

Is the statistical analysis and its interpretation appropriate?

Yes

Are the conclusions drawn adequately supported by the results presented in the review?

Partly

Reviewer Expertise:

Saba Hinrichs-Krapels: Systematic literature reviews, public policy analysis. Palok Biswas: Model based decision making, artificial intelligence, climate justice and climate economics.

I confirm that I have read this submission and believe that I have an appropriate level of expertise to confirm that it is of an acceptable scientific standard.

# Reviewer report: Reviewer response for version 2

**DOI:** 10.21956/openreseurope.17995.r35546

**Published:** 2023-10-24

**Authors:** Eric Brandstedt

**Affiliations:** 1 Lund University, Lund, Sweden

I  approve the revisions and I have no further comments. My verdict to approve.

Are sufficient details of the methods and analysis provided to allow replication by others?

Yes

Are the rationale for, and objectives of, the Systematic Review clearly stated?

Yes

Is the statistical analysis and its interpretation appropriate?

Yes

Are the conclusions drawn adequately supported by the results presented in the review?

Partly

Reviewer Expertise:

NA

I confirm that I have read this submission and believe that I have an appropriate level of expertise to confirm that it is of an acceptable scientific standard.

# Reviewer report: Reviewer response for version 1

**DOI:** 10.21956/openreseurope.16983.r32755

**Published:** 2023-06-19

**Authors:** Eric Brandstedt

**Affiliations:** 1 Lund University, Lund, Sweden

This is a systematic review of interdisciplinary literature around climate justice. It is clearly structured, with a solid method (barring the oversight described below) and informative summaries of the reviewed articles. The overall objective of the article – to connect this literature to research on policy processes – is well chosen, relevant and important.

 However, a major flaw of the manuscript in its present form is that it overlooks a centrally relevant field of research. I'm referring to the field of study known as "climate justice" or "climate ethics" to which normative political theorists and philosophers have contributed since the early 1990s (at first, in the 90s, with just a few contributions by e.g., Henry Shue, and then very many contributions since 2005 and onwards, by e.g., Simon Caney, Ed Page, Stephen Gardiner and many others). An overview of this research can be found here: https://plato.stanford.edu/entries/justice-climate/ (Caney 2021). I'm amazed that the authors have missed this whole field of research on climate justice in a systematic review on precisely this topic. In any case, this is something that must be addressed.

 In reviewing the field of climate justice which I've just referred to, the authors will also note that the question about the gap between normative ideals of climate justice and the political reality around climate policy-making has been one of the topics that has been addressed there. This has generally been discussed under the heading of "non-ideal climate justice" (I reviewed that discussion in Brandstedt 2019, see also the contributions in Heyward and Roser 2016, and Caney 2016).

 Another point is that it would be good for the authors to also reflect on what is the objective of theorising climate justice. The review now seems to assume that the aim of this kind of theorising is to provide input to policy design, but that is actually just one of several objectives that are possible. Other functions of climate justice theorising could be to evaluate practices, to identify injustices, to articulate claims and grievances of activists and affected parties, and to challenge taken-for-granted assumptions and concepts (while recognising that the theoretical products produced cannot simply be implemented in political practices). Fergus Green and I (2021) discuss various roles for climate justice theorists and argue for more "engagement" with practices, which we argue could e.g., be done by working closer to policy processes (and we also give examples of climate justice theorists who have done this already, e.g., Andrew Light and Catriona McKinnon). That is not to say that there is no room for improvement here. On the contrary, I very much agree with the overall conclusion of the review article that climate justice theorists in general tend to treat policy processes as a black box and I also agree that it would be fruitful to engage further with research (and not only practice) on how policy processes work.

**References**

 Brandstedt, Eric. 2019. “Non-Ideal Climate Justice.” *Critical Review of International Social and Political Philosophy* 22(2): 221–34. https://doi.org/10.1080/13698230.2017.1334439.

 Caney, Simon. 2016. “The Struggle for Climate Justice in a Non‐Ideal World.” *Midwest Studies In Philosophy* 40(1): 9–26. http://onlinelibrary.wiley.com/doi/10.1111/misp.12044/full.

 Caney, Simon. 2021. “Climate Justice.” *Stanford Encyclopedia of Philosophy*. (Winter 2021 Edition), Edward N. Zalta (ed.), URL = https://plato.stanford.edu/archives/win2021/entries/justice-climate/.

 ﻿Heyward, C., & Roser, D. (Eds.). (2016). Climate Justice in a Non-Ideal World. Oxford: Oxford University Press

 Green, Fergus, and Eric Brandstedt. 2021. “Engaged Climate Ethics.” *Journal of Political Philosophy* 29(4): 539–63.

Are sufficient details of the methods and analysis provided to allow replication by others?

Yes

Are the rationale for, and objectives of, the Systematic Review clearly stated?

Yes

Is the statistical analysis and its interpretation appropriate?

Yes

Are the conclusions drawn adequately supported by the results presented in the review?

Partly

Reviewer Expertise:

I work on climate justice, climate ethics, intergenerational justice, human rights, and political philosophy in general.

I confirm that I have read this submission and believe that I have an appropriate level of expertise to confirm that it is of an acceptable scientific standard, however I have significant reservations, as outlined above.

## References

[1] : Non-ideal climate justice. *Critical Review of International Social and Political Philosophy* .2019;22(2) : 10.1080/13698230.2017.1334439 221-234 10.1080/13698230.2017.1334439

[2] : The Struggle for Climate Justice in a Non-Ideal World. *Midwest Studies In Philosophy* .2016;40(1) : 10.1111/misp.12044 9-26 10.1111/misp.12044

[3] : Climate Justice in a Non-Ideal World. *Oxford: Oxford University Press* .2016;

[4] : Engaged Climate Ethics*. *Journal of Political Philosophy* .2021;29(4) : 10.1111/jopp.12237 539-563 10.1111/jopp.12237

# Response

**Published:** 2023-09-19

**Authors:** Paul Cairney

**Affiliations:** University of Stirling, UK

We thank Brandstedt (2023) for a highly constructive review that prompts us to address key aspects of our article. We summarise the key points as follows:

 1. Address the omission of a field of research – ‘climate justice’ or ‘climate ethics’ – that should be seen as essential.

 Response: We clarify our scope in the Abstract, to describe inclusion in relation to policy concepts/ theories. We also make a more explicit statement about the limitations of this review in the Methods section. We note that our inclusion rules produced the problem described by Brandstedt: ‘For example, Brandstedt’s (2023) peer review notes that our search rules – requiring engagement with policy concepts - lead to the omission of ‘the field of study known as "climate justice" or "climate ethics" to which normative political theorists and philosophers have contributed since the early 1990s. Further, we confirm, after reviewing the sample of texts described by Brandstedt (2023), that such research would generally not be included in our review’. Stephan and Cairney conducted this additional review process to produce this new statement. For example, Caney’s (2021) overview does not refer to any concepts or sources described in our list, or discuss policymaking as a process worthy of investigation in its own right. A more relevant candidate is Caney’s (2016) ‘Political Institutions for the Future: A Fivefold Package’ (in Institutions for Future Generations, ed. Axel Gosseries and Iñigo González Ricoy. Oxford: Oxford University Press) since it cites Bachrach and Baratz on power and framing (a classic in the policy theories field). However, this example also demonstrates the practical limits to inclusion, in which we would need to search entire books for fleeting references to individual references to a relevant text (without finding a more meaningful engagement with a policy theory or literature).

 2. When addressing the omission of ‘climate justice’ or ‘climate ethics’, note that it has already engaged with ‘the gap between normative ideals of climate justice and the political reality around climate policy-making’.

 Response: In Limitations, we describe what is missing from our review’s coverage as a result of our omission of the literature: ‘Nevertheless, as Brandstedt’s (2023) peer review notes, our search rules excluded the direct examination of ‘climate ethics’ research that engages with ‘normative ideals of climate justice’ and ‘the political reality around climate policy-making’ (although many of the included articles engage with those concerns, while some - such as Sovacool *et al*., 2017 – cite Caney and colleagues directly). As a result, we have engaged relatively sparingly with key concerns in the wider research field, such as how to conceptualise climate justice and injustice (an important exercise in its own right)’. This new paragraph also addresses point 3 below. We also note that our interest is not only ‘political reality’ but also policymaking reality. In other words, when we describe the use of policy theories and concepts the aim is to examine the importance of policymaking in its own right, rather than simply a reflection of political contestation (as described in relation to Review 1, point 5).

 3. Reflect on the multiple roles that could be performed while theorising climate justice (engaging with policy processes is one of many useful aims).

 Response: we agree wholeheartedly that we should not seek to describe climate justice studies as only performing a policy analysis role. Rather, our aim is to engage with studies which appear to be seeking policy concept/ theory insights to that end (in other words, our review is of an important subset of climate justice research). We use the new paragraph in Limitations to address points 2 and 3.

# Reviewer report: Reviewer response for version 1

**DOI:** 10.21956/openreseurope.16983.r31105

**Published:** 2023-06-01

**Authors:** Saba Hinrichs-Krapels, Palok Biswas

**Affiliations:** 1 Faculty of Technology, Policy and Management, Technische Universiteit Delft, Delft, South Holland, The Netherlands; 2 Delft University of Technology, Delft, South Holland, The Netherlands

This research article presents a comprehensive review of the diverse climate justice literature, insightfully structured around ecological modernization and civic environmentalism policy theories. It serves as a valuable starting point for understanding climate justice policy requirements and the gaps between goals and actual policies.

 Overall the manuscript is well written and is a valuable contribution to the literature. Specific feedback is given below:

**Under Methods/Rationale/Engagement with previous reviews:** The authors describe their methods thoroughly and make reference to Kuckertz and Block’s (2021) which I believe is adequate for this review. The OSF link has the necessary documentation to study their search strategy (PRISMA flowchart, checklist, etc).

 The authors refer to the Horizon 2020 project to which this review belongs, to set context, and mention other reviews conducted (some of the authors themselves). While this helps in understanding the context for this review and its general motivation, it does not provide a rationale for why this new review is needed. Some sentences motivating this (particularly with references to gaps in previous reviews) would help situate this review in the wider literature. This also follows the guidance from Kuckertz and Block’s (2021) to which the authors refer (guiding question 2: 2. Does the SLR acknowledge previous literature reviews (including meta-analyses)?).

 Given the ‘high contestation’ on definitions for justice, fairness and equity found in the articles, I would suggest the article would benefit from making transparent what definitions the authors themselves used in identifying these concepts for each study they reviewed. This would be best placed after introducing the review’s overall guiding question: “How does equity research use policy theory to understand policymaking?” (e.g. did the authors interpret other authors’ uses of these terms as relating to equity/justice/fairness, or only search for these keywords?)

**Results:** Although the text and analysis are rich, and the authors clearly discuss the included articles in depth, there is some inconsistency in the two questions that guided the analysis (in the methods section, which focussed on ‘describing policymaking processes’ and ‘transferrable lessons’) and the many questions answered in the results. The results focus on context, definitions of climate justice, changes in policy sought, changes in policy found, use of policy theory. The guiding question on ‘transferrable lessons’ is therefore hidden and not explicitly answered. It would help if the authors either introduce these guiding questions up front (if indeed they were guiding the analysis), or, if these themes were truly grounded and emerging from the including articles, a better relation to their initial guiding questions, along with a summary at the start of the results to guide these themes and patterns would be helpful. The text otherwise risks coming across as inconsistent and meandering from its objectives, which is probably not the intention.

*On uncertainties with climate justice:* The authors claim that the requirements for climate justice are clear. In reality, models used for policy recommendation are fraught with deep uncertainty, making the requirements less clear. Firstly, climate sensitivity to CO2 concentrations is uncertain, resulting in differing projections for future temperature rise ^1^. Secondly, economic uncertainty in the models used for climate policies is significant, and the cost of abatement is primarily driven by this economic uncertainty, indicating the need for further exploration of uncertainties rather than strict policy requirements ^4^. Furthermore, how temperature rise affects economic growth is also uncertain, with different estimates of climate damages projecting varying economic damages ^3^.

 These modelling uncertainties, known as epistemic uncertainties, raise questions about the extent of mitigation and adaptation necessary. This epistemic uncertainty also leads to epistemic normative uncertainty. Given the uncertain temperature rise and damage projections, epistemic normative uncertainty arises when policymakers are unsure how to value present versus future generations. This normative uncertainty debate between Nordhaus and Stern illustrates this: Nordhaus argues for adaptation with a discount rate of 5.5%, while Stern advocates for mitigation with a discount rate of 1.4% ^2^.

 The paper could benefit from addressing these concerns regarding climate and economic uncertainties and their implications on policy requirements in the "Addressing Uncertainty and Ambiguity" subsection.

**Discussion:** The authors start this section with stating that three core policy theory insights “should guide further interdisciplinary research in relation to climate justice”. Although I do not necessarily disagree with the choices, it is unclear how the authors concluded that these theories should be the guiding ones in this context. For example, there are also studies that examine the behavioural competency assumptions of policy addressees and relate these to the policy levers to combat any public policy process. ^5^

**Minor point:** A minor correction is needed on page 12 in the Sovacool's (2021:1-2) paragraph, where the words 'showswhat' should have a space between them.

Are sufficient details of the methods and analysis provided to allow replication by others?

Yes

Are the rationale for, and objectives of, the Systematic Review clearly stated?

Yes

Is the statistical analysis and its interpretation appropriate?

Yes

Are the conclusions drawn adequately supported by the results presented in the review?

Partly

Reviewer Expertise:

Saba Hinrichs-Krapels: Systematic literature reviews, public policy analysis. Palok Biswas: Model based decision making, artificial intelligence, climate justice and climate economics.

We confirm that we have read this submission and believe that we have an appropriate level of expertise to confirm that it is of an acceptable scientific standard, however we have significant reservations, as outlined above.

## References

[1] : Robust abatement pathways to tolerable climate futures require immediate global action. *Nature Climate Change* .2019;9(4) : 10.1038/s41558-019-0426-8 290-294 10.1038/s41558-019-0426-8

[2] : Governing climate risks in the face of normative uncertainties. *WIREs Climate Change* .2020;11(5) : 10.1002/wcc.666 10.1002/wcc.666

[3] : New damage curves and multimodel analysis suggest lower optimal temperature. *Nature Climate Change* .2023;13(5) : 10.1038/s41558-023-01636-1 434-441 10.1038/s41558-023-01636-1

[4] : The costs of achieving climate targets and the sources of uncertainty. *Nature Climate Change* .2020;10(4) : 10.1038/s41558-020-0732-1 329-334 10.1038/s41558-020-0732-1

[5] : Comparing Behavioral Assumptions of Policy Tools: Framework for Policy Designers. *Journal of Comparative Policy Analysis: Research and Practice* .2020;22(6) : 10.1080/13876988.2020.1808465 498-520 10.1080/13876988.2020.1808465

# Response

**Published:** 2023-09-19

**Authors:** Paul Cairney

**Affiliations:** University of Stirling, UK

We thank Hinrichs-Krapels and Biswas (2023) for their constructive suggestions to improve our article. In each case, they prompt us to justify, explain, and/ or clarify our aims and choices. We summarise their required changes, and our responses, as follows:

**Under Methods/Rationale/Engagement with previous reviews**

 1. Provide a more specific rationale for the review, ‘particularly with references to gaps in previous reviews’.

 Response: we perform this task in the Introduction and Methods sections. The new introductory text identifies the lack of an overall assessment of policy concept/ theory usage in climate justice research: ‘Previous review articles have contributed separately to key elements of this task, to identify how climate researchers: conceptualise justice or injustice (Malloy and Ashcraft, 2020; Sokolowski and Heffron, 2022; Pellegrini-Masini *et al*., 2020), establish feasible pathways to policy change (Sovacool *et al*., 2020; Werners *et al.*, 2021), describe the injustices that can result from climate-related policy changes (Sovacool, 2021; Kock and Verholt, 2020), identify the policy concepts and theories useful to climate research (Huitema *et al.*, 2016), and use key theories as part of a framework to assess climate governance (Burch *et al.*, 2019). Here, we consolidate and build on such work to provide an overall assessment of the use of policy concepts and theories to inform research on climate justice policy and policymaking’.   The new text in Methods/ Rationale states: ‘We seek to understand the use of policy concepts and theories in climate justice research. These theories are essential to research (1) identifying requirements for policy and policymaking change, but (2) unable to fully appreciate how policy processes work’

 2. Clarify how we defined ‘justice, fairness, and equity’ when seeking articles to review.

 Response: we note in the original draft that researchers (1) often seem to use these general terms interchangeably, and  (2) identify similar categories in relation to recognitional, procedural, distributional justice. In this revision we clarify that we (1) use ‘equity’ or ‘justice’  as part of an initial keyword search, without including or excluding on the basis of how researchers define these terms, and (2) explore how researchers define these terms, rather than seeking to consolidate research with one overarching definition: “We sought to understand how climate researchers define terms like ‘justice’ or ‘equity’ rather than to include/ exclude based on our definition”.

**Results**

 3. Clarify the role of the initial guiding questions (e.g. on transferable lessons) and the subsequent themes that emerged.

 Response: we revise the ‘Research/ guiding questions’ text to reduce confusion and emphasise immersion in the field. The previous wording was describing the approach taken to the wider project, at the expense of an accurate description of this particular review. Now, we note that ‘However, we have found in previous reviews that most articles do not provide substantive answers to such questions … Here, we focus on making sense of research on the inequalities that relate to climate change, based more on immersion in the field than trying to stretch the results to fit our previous categories and questions’.

 4 **. **Clarify the claim in our paper that ‘requirements for climate justice are clear’ in the context of high epistemic uncertainty (regarding scientific and economic models used to generate recommendations).

 Response: we amend the text to clarify that we were not dismissing epistemic uncertainty. Rather, we are distinguishing between relative clarity regarding (1) what scholars would prefer to happen versus (2) how to make it happen in complex policymaking systems. Our small changes include replacing ‘clear requirement’ with ‘general requirement’ to avoid misinterpretation in the Introduction. We also refer directly to this referee advice in Results/ The IPCC: “These testimonials are important in relation to two connected issues. First, there are inevitable ‘epistemic uncertainties’ in relation to predicting climate change and the likely impact of policy (Hinrichs-Krapels and Biswas, 2023).  Second, the exploitation of uncertainty is a regular feature of partisan debates and climate denial …”. We suggest that this placement of the new text is more suited to the task at hand than the amendment of the ‘Addressing uncertainty’ section. 

**Discussion**

 5. Explain why these ‘three core policy theory insights’ should be the main focus.

 Response: we appreciate this prompt to explain our approach properly. To that end, we add a short section at the beginning of the Discussion to make the case for policy concepts and theories in general, then clarify that the three concepts relate to our synthesis of the broad field. ‘Engagement with policy concepts and theories is important because policy processes are essential objects of study in their own right. Put simply, climate justice research may engage with political reality, but it also needs to engage with the realities of policymaking or policy processes. Empirical studies, guided by policy concepts and theories, help to identify the routine patterns or dynamics of policymaking that are present regardless of the specific politics of the day. They identify features of policymaking that are general rather than specific to climate policy, such as a tendency towards limited policy change, an in-built need for policymakers to ignore most issues and evidence, and a complex policymaking system or environment over which they have limited knowledge and even less control. In that context, we present the following three core insights based on our synthesis of policy theories and empirical studies on the nature of policy change, bounded rationality, and policymaking complexity. We argue that all three broad insights should guide further interdisciplinary research in relation to climate justice and policymaking, but only find meaningful engagement with one (regarding bounded rationality).’ As described, this broad approach does not rule out the specific studies or concepts described in review 1.
